# Upregulation of RIG‐I is Critical for Responsiveness to IFN‐α Plus Anti‐PD‐1 in Colorectal Cancer

**DOI:** 10.1002/cam4.70802

**Published:** 2025-03-21

**Authors:** Haihang Nie, Shilin Fang, Rui Zhou, Yifan Jia, Jingkai Zhou, Yumei Ning, Yali Yu, Yuntian Hong, Fei Xu, Qiu Zhao, Jiayan Nie, Fan Wang

**Affiliations:** ^1^ Department of Gastroenterology Zhongnan Hospital of Wuhan University Wuhan China; ^2^ Hubei Provincial Clinical Research Center for Intestinal and Colorectal Diseases Wuhan China; ^3^ Hubei Key Laboratory of Intestinal and Colorectal Diseases Zhongnan Hospital of Wuhan University Wuhan China; ^4^ Department of Infectious Diseases Zhongnan Hospital of Wuhan University Wuhan China; ^5^ Department of Pain Renmin Hospital of Wuhan University Wuhan China

**Keywords:** anti‐PD‐1, bioinformatic analysis, colorectal cancer, combinatorial immunotherapy, interferon‐α, RIG‐I

## Abstract

**Backgrounds:**

Immunotherapy is a promising and effective approach that has achieved significant curative effects in colorectal cancer (CRC). Recently, retinoic acid‐inducible gene I (RIG‐I) has been shown to play a critical role in tumor immunity. However, the correlation between RIG‐I and immunotherapy in CRC remains unclear.

**Methods:**

RIG‐I expression was measured in CRC and normal samples based on analysis of the public databases, a tissue microarray, and CRC cell lines. The correlation between RIG‐I and immune microenvironment was explored using well‐established biological algorithms and in vitro and in vivo experiments.

**Results:**

We discovered that RIG‐I expression was downregulated in CRC compared with normal samples. The bioinformatic algorithms indicated that high RIG‐I‐expressing samples showed a positive correlation with IFN‐α response and enrichment of antitumor immune cells, especially CD8+ T cells. Furthermore, knockdown of RIG‐I expression efficiently reduced the cell death, STAT1 phosphorylation, and CXCL10/11 expression induced by IFN‐α in CRC cells. Finally, an in vivo study showed that the infiltration of CD3+ CD8+ T cells was significantly decreased in the RIG‐I knockout group. An animal model further confirmed that the inhibition of tumor growth induced by IFN‐α plus anti‐PD‐1 therapy was dependent on RIG‐I expression.

**Conclusion:**

RIG‐I is a promising biomarker for CRC immunotherapy, which provides a novel concept for combinatorial immunotherapy.

## 1 Introduction

Colorectal cancer (CRC) is a malignant tumor that ranks as the second leading cause of cancer‐related mortality worldwide, posing a significant global health burden [1, 2]. Despite substantial advancements in conventional therapies, including surgical resection, chemotherapy, and radiotherapy, the overall prognosis of CRC remains poor due to recurrence, distant metastasis, and chemoresistance [3, 4]. Therefore, there is an urgent need to develop novel therapeutic strategies to improve patient survival.

Immunotherapy, particularly immune checkpoint inhibitors (ICIs), has shown significant clinical benefits in treating microsatellite instability‐high (MSI‐H) colorectal cancer (CRC) and other cancers [5, 6]. MSI is marked by changes in microsatellite length and is often linked to deficient mismatch repair (dMMR) proteins [7, 8]. Pembrolizumab and nivolumab have been approved by the FDA for second‐line treatment of dMMR/MSI‐H CRC [9, 10]. In addition, interferon‐α (IFN‐α), a widely used treatment, enhances CD8+ T cell function and exerts antitumor effects [11]. It has been used to treat several cancers, including CRC, melanoma, renal cell carcinoma, and hepatocellular carcinoma (HCC) [12, 13]. However, not all CRC patients respond to immunotherapy due to intrinsic or acquired resistance [14]. Therefore, identifying reliable biomarkers to predict responses to immunotherapy is crucial for improving treatment outcomes.

Retinoic acid‐inducible gene I (RIG‐I) was originally identified as a cytosolic pattern recognition receptor that binds viral RNA and plays a key role in activating antiviral immune responses [15]. RIG‐I activation can directly trigger inflammasomes, leading to the death of virus‐infected cells [16, 17]. Additionally, RIG‐I induces the expression of proinflammatory cytokines as an interferon‐stimulated gene (ISG), initiating an immune response to eliminate infected cells [18, 19]. Beyond its antiviral function, RIG‐I has been shown to have antitumor effects in various cancers. For example, in hepatocellular carcinoma (HCC), low RIG‐I expression correlates with poor prognosis and resistance to IFN‐α treatment [19]. Our recent study also revealed that RIG‐I downregulation contributes to resistance to IFN‐α‐induced apoptosis in melanoma cells [14]. RIG‐I activation enhances innate immune responses in the tumor microenvironment, boosting NK cell‐mediated cytotoxicity and the antigen‐presenting functions of macrophages and dendritic cells [20, 21]. These findings suggest RIG‐I as a potential biomarker for predicting cancer patients' responses to immunotherapy. However, its relationship with the immune microenvironment and immunotherapy response in CRC has not been explored.

In this study, we examined RIG‐I expression differences between CRC and normal colonic tissues using public databases, human CRC samples, and specific cell lines. We also used bioinformatics algorithms to analyze how RIG‐I expression correlates with the immune microenvironment and immunotherapy responses. Finally, we validated these findings using an in vivo animal model. Our results offer new insights into the role of RIG‐I in CRC immunotherapy and may help identify predictive biomarkers for therapeutic response.

## 2 Methods and Materials

### 2.1 CRC Patients and Public Data Preparation

**FIGURE 1:**
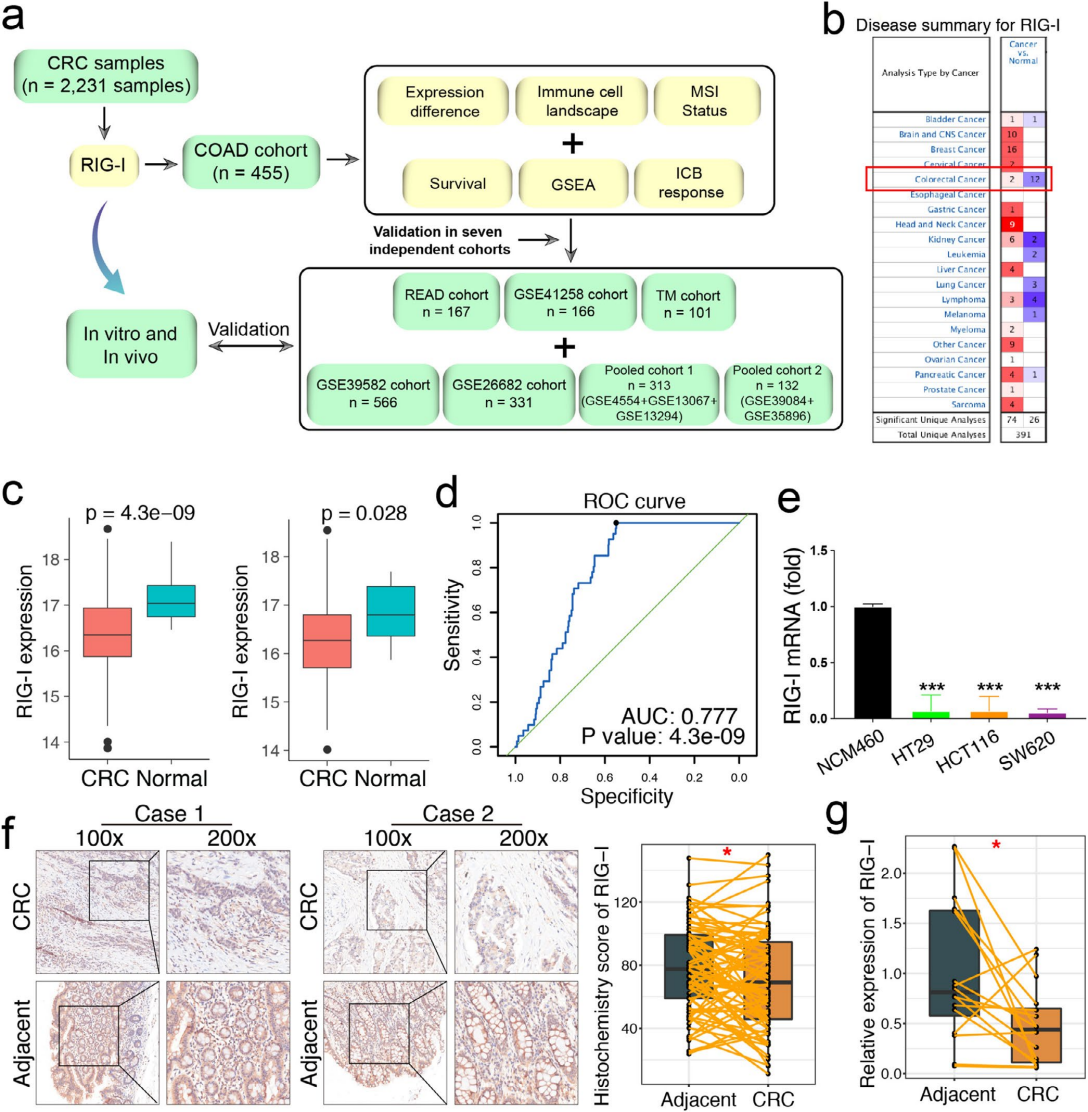
RIG‐I expression is downregulated in CRC. (a) Overview of the whole procedures of the study. (b) ONCOMINE database shows the mRNA level of RIG‐I in colorectal cancer and normal tissues. The red box shows 12 datasets with statistically significant mRNA downexpression (blue) or 2 overexpressions (red) of RIG‐I under the threshold of fold change > 1.5 and *p* < 0.05. (c) The expression differences of RIG‐I in CRC in TCGA. (d) The ROC curve shows the diagnostic efficiency of the RIG‐I in the TCGA‐COAD dataset (normal vs. CRC, AUC = 0.777). (e) Total RNA of NCM460, HT29, HCT116, and SW620 was collected and reverse transcribed. Then, RIG‐I mRNA expression was analyzed by RT‐qPCR. (f) Representative immunohistochemical staining of RIG‐I in CRC and paired adjacent tissues. The corresponding histochemistry score was also shown. (g) Quantitative qPCR analysis showed that RIG‐I expression was downregulated in 15 CRC tissues compared to paired adjacent tissues. Each experiment was repeated independently at least three times. Data were presented as the mean ± SEM. *p < 0.05, **p < 0.01, ***p < 0.001, and ****p < 0.0001 versus the control group. AUC, Area under the curve; COAD, colon adenocarcinoma; CRC, colorectal cancer; READ, rectum adenocarcinoma; ROC, receiver operating characteristic.

Figure 1a provides an overview of the study procedure. The upper quartile fragments per kilobase of transcript per million mapped reads (FPKM‐UQ) data from The Cancer Genome Atlas (TCGA) colon adenocarcinoma (COAD) cohort was used as the training dataset (https://xenabrowser.net/datapages/). We also selected six independent cohorts for validation: TCGA rectum adenocarcinoma (READ), GSE39582, GSE41258, GSE26682, pooled cohort 1, and pooled cohort 2. Pooled cohort 1 included GSE13067, GSE13294, and GSE4554, while pooled cohort 2 contained GSE35896 and GSE39084. To minimize batch effects in the pooled cohorts, we applied the “sva” R package [22] and visualized the results using principal component analysis (PCA) (Figure S1). Detailed information about the cohorts is summarized in Table S1, and the clinical data for all seven cohorts is provided in Table S2. Probe annotation was carried out based on the respective platform annotation files.

Additionally, 15 CRC and 15 adjacent normal tissue samples were collected from patients at Zhongnan Hospital of Wuhan University. A tissue microarray (TMA) for evaluating RIG‐I protein expression was obtained from the Biobank of the National Engineering Center for Biochip in Shanghai (National Human Genetic Resources Sharing Service Platform, No. 2005DKA21300). Detailed protocols for bioinformatics analyses and experimental procedures are available in the online supplemental file.

### 2.2 Cell Culture and Reagents

Human CRC cell lines (HT29, SW620, and HCT116), a human colonic epithelial cell line (NCM460), and a murine CRC cell line (MC38) were obtained from the China Center for Type Culture Collection (Beijing, China). All cell lines were maintained in RPMI 1640 medium (Hyclone, USA) supplemented with 10% fetal bovine serum (Gibco, Australia) and 1% penicillin–streptomycin at 37°C in a humidified incubator with 5% CO_2_. Human and murine recombinant IFN‐α were purchased from Novoprotein, and the in vivo murine anti‐PD‐1 antibody (BE0146, USA) was obtained from BioXCell.

### 2.3 Creation of a Stable Knockout Cell Line Using CRISPR Technology

MC38 cells were used to generate stable RIG‐I and MAVS knockout cell lines to validate immunotherapeutic effects, as previously described [23]. Briefly, MC38 cells were transfected with PX459 (Addgene) containing sgRNA targeting murine RIG‐I, MAVS, or green fluorescent protein (GFP) as a control. After 24 h, puromycin‐resistant cells were selected and seeded into 96‐well plates for clonal expansion. Knockout of RIG‐I and MAVS was confirmed by western blotting, and the cells were subsequently cultured for further analysis. The corresponding primer sequences are provided in Table S4.

### 2.4 Animal Model

MC38 cells (1 × 10^6^/ml) were subcutaneously injected into the right flanks of C57BL/6J mice. Seven days postinjection, the mice were randomly assigned to four groups: control, IFN‐α, anti‐PD‐1, and IFN‐α + anti‐PD‐1. Intratumoral injections of anti‐PD‐1 antibody (250 μg per mouse) or IFN‐α (2 × 10^5^ U/mouse) were administered every 2 days until the end of the observation period (Day 25). Control groups received equivalent doses of IgG or PBS following the same schedule. Tumor volume (mm^3^) was measured every other day and calculated using the formula: V = a × b^2^/2.

### 2.5 Statistical Analysis

All experiments were performed at least three times. Data are presented as the mean ± SEM and were analyzed using unpaired or paired Student's *t*‐tests, as well as one‐way or two‐way ANOVA. Statistical analyses were conducted using GraphPad Prism 7.0 and R 3.6.1. A *p*‐value < 0.05 was considered statistically significant.

## 3 Results

### 3.1 RIG‐I Expression is Downregulated in CRC

Through a comprehensive analysis of CRC mRNA data from TCGA and the public ONCOMINE database (https://www.oncomine.org/), we found that RIG‐I expression was significantly downregulated in CRC compared with adjacent normal tissues (Figure 1b,c). Receiver operating characteristic (ROC) curve analysis demonstrated that RIG‐I exhibited strong diagnostic potential for CRC in the COAD cohort (AUC = 0.77, *p* = 4.3 × 10^−9^, Figure 1d). Compared with the normal intestinal epithelial cell line NCM460, RIG‐I expression was significantly reduced in CRC cell lines (HT29, HCT116, and SW620) (Figure 1e). Additionally, immunohistochemical analysis revealed a lower histochemistry score for RIG‐I in tumor samples than in adjacent normal tissues (Figure 1f). Consistently, RIG‐I mRNA levels were also significantly decreased in CRC samples (Figure 1g). Furthermore, we investigated the prognostic significance of RIG‐I in CRC. However, no significant correlation was observed between RIG‐I expression and prognosis across the seven cohorts analyzed (Figure S2). Taken together, these findings confirm that RIG‐I expression is downregulated in CRC.

### 3.2 High RIG‐I Expression is Associated With Microsatellite Instability and Distinct Mutational Patterns in CRC

**FIGURE 2:**
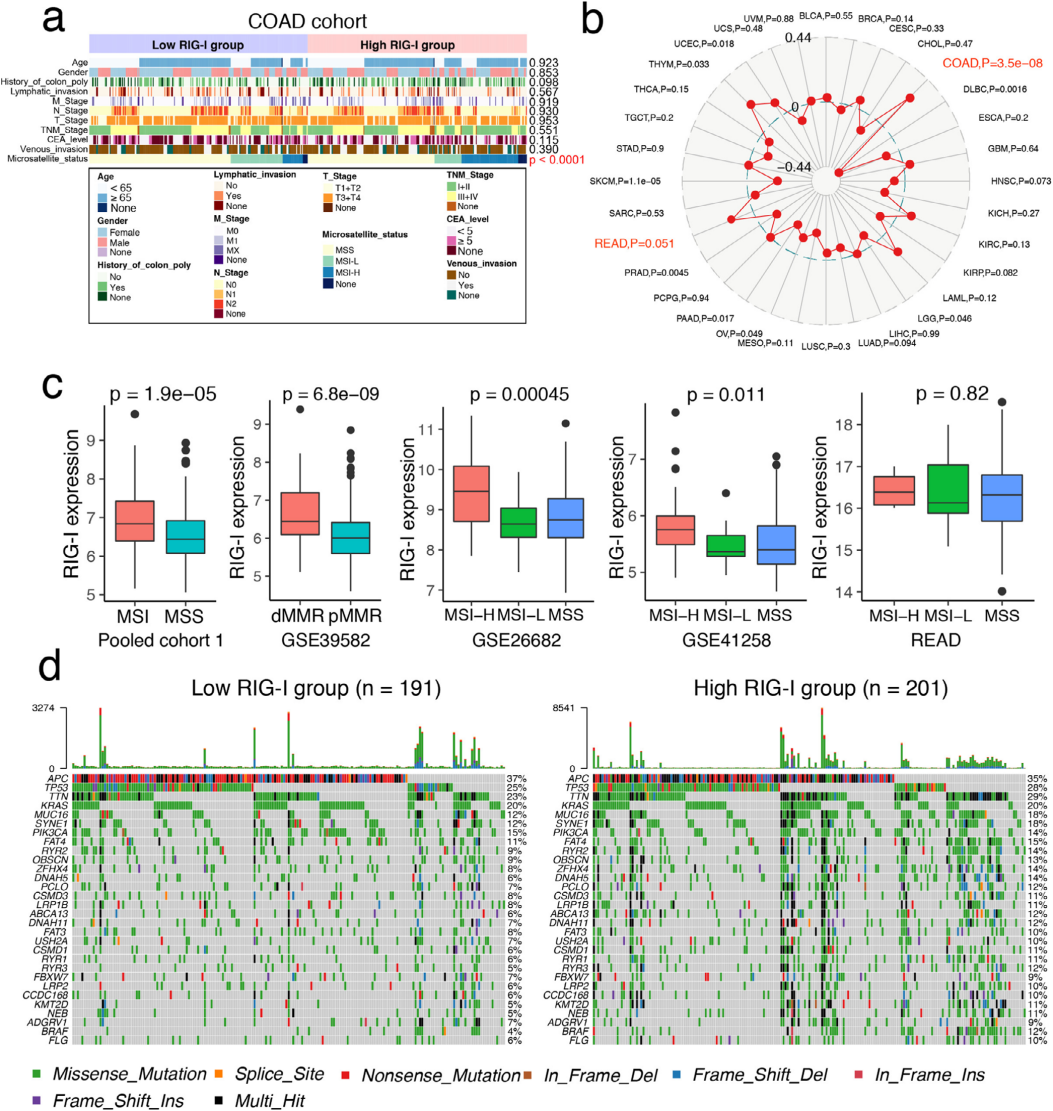
High RIG‐I expression is associated with MSI status and distinct mutational patterns in CRC. (a) Correlation of RIG‐I expression with clinical phenotypes in the COAD cohort. (b) Spider plot showing the correlation of RIG‐I expression with MSI status across all TCGA tumors. (c) The difference in MSI and MSS between high‐ and low‐RIG‐I groups based on the pooled cohort 1, GSE39582, GSE26682, GSE41258, and READ cohorts. (d) The waterfall plots showed the top 30 gene alterations in the RIG‐I high and low expression groups, respectively. *p < 0.05, **p < 0.01, ***p < 0.001, and ****p < 0.0001 versus control group. COAD, colon adenocarcinoma; CRC, colorectal cancer; dMMR, deficient mismatch repair; MSI, microsatellite instability; MSS, microsatellite stability; pMMR, proficient mismatch repair.

We next investigated the correlation between RIG‐I expression and clinical characteristics. No significant associations were found with clinicopathological features (Figures 2a, S3 and Table 1). The proportion of the C2 subtype (dMMR) was higher, while the C1 subtype (ImmuneDown) was lower in the RIG‐I high‐expression group [24]. Additionally, upregulation of RIG‐I was more common in the proximal (right) colon (Figure S3a).

**TABLE 1:** The characteristics of patients in the tissue microarray.

Variables	RIG‐I expression			*p*
	Total (*n* = 101)	High (*n* = 51)	Low (*n* = 50)	
Gender				
Male, (%)	50 (49.5)	27 (52.9)	23 (46.0)	0.485
Female, (%)	51 (50.5)	24 (47.1)	27 (54.0)	
Age				
< 65 years, (%)	38 (37.6)	21 (41.2)	17 (34.0)	0.457
≥ 65 years, (%)	63 (62.4)	30 (58.8)	33 (66.0)	
Grade				
≤ Grade II, (%)	84 (83.2)	43 (84.3)	41 (82.0)	0.690
> Grade II, (%)	16 (15.8)	7 (13.7)	9 (18.0)	
NA, (%)	1 (0.9)	1 (2.0)	0 (0.0)	
Tumor volume				
≤ 20 cm^3^, (%)	44 (43.7)	23 (45.1)	21 (42.0)	0.842
> 20 cm^3^, (%)	56 (55.4)	28 (54.9)	28 (56.0)	
NA, (%)	1 (0.9)	0 (0)	1 (2.0)	
T stage				
T1 + 2, (%)	6 (5.9)	2 (3.9)	4 (8.0)	0.061
T3, (%)	75 (74.3)	34 (66.7)	41 (82.0)	
T4, (%)	19 (18.8)	14 (27.4)	5 (10.0)	
N stage				
N0, (%)	61 (60.4)	29 (56.9)	32 (64.0)	0.727
N1, (%)	30 (29.7)	16 (31.4)	14 (28.0)	
N2, (%)	10 (9.9)	6 (11.7)	4 (8.0)	
M stage				
M0, (%)	100 (99.0)	50 (98.0)	50 (100.0)	0.505
M1, (%)	1 (1.0)	1 (2.0)	0 (0)	
TNM stage				
I + II, (%)	61 (60.4)	29 (56.9)	32 (64.0)	0.463
III + IV, (%)	40 (39.6)	22 (43.1)	18 (36.0)	

Abbreviation: NA, not available.

We also found that RIG‐I expression was significantly higher in MSI‐H samples from the COAD cohort (Figure 2a) and most strongly associated with MSI status in COAD across TCGA tumors (Figure 2b). This was confirmed in five other independent CRC cohorts (Figure 2c). However, no significant correlation was found in the READ cohort, which contained only six MSI samples (Figure 2c). Our findings align with those of Wang et al., who reported increased RIG‐I expression in MSI CRC across three datasets [25]. Thus, our data complement Wang et al.'s study. We further analyzed somatic mutations in the COAD cohort to determine whether RIG‐I expression groups had different mutation profiles. A waterfall plot revealed that the top 30 genes had a higher mutation rate in the high RIG‐I group (Figure 2d). Validation in three independent cohorts showed that high RIG‐I expression was associated with the BRAF V600E mutation, while no significant differences were found for KRAS, TP53, and PI3KCA mutations (Figure S4). These findings suggest that high RIG‐I expression is linked to a higher mutational burden and MSI occurrence.

### 3.3 RIG‐I Expression Positively Correlates With IFN Responses and Infiltrating Immune Cells in CRC

**FIGURE 3:**
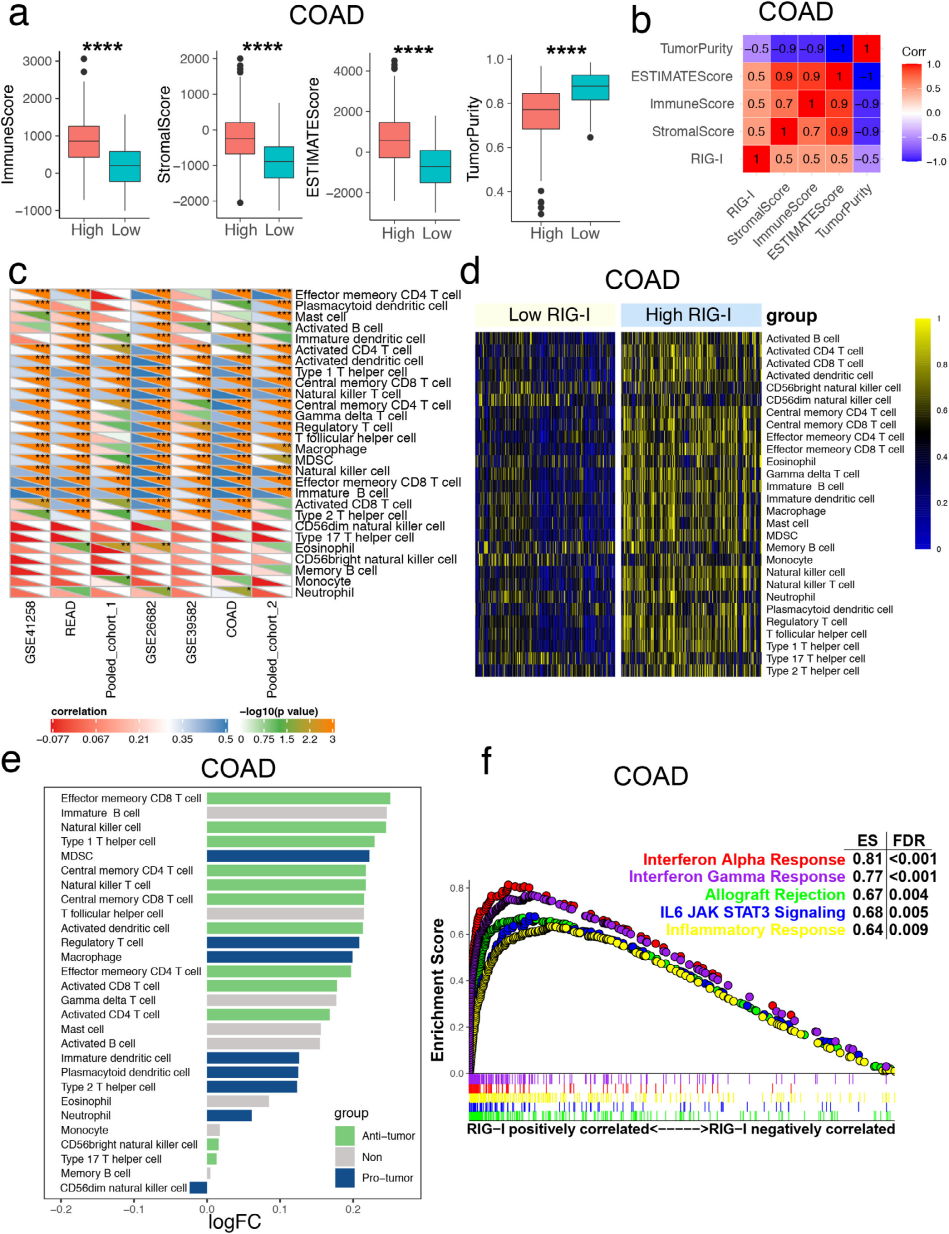
High expression of RIG‐I promotes the activation of antitumor immune cells based on the immune cell landscape. (a) The box plot indicated the difference in ImmuneScore, StromalScore, ESTIMATEScore, and TumorPurity between high‐ and low‐RIG‐I groups of the COAD cohort. (b) Heatmap showed the correlation between RIG‐I expression and ImmuneScore, StromalScore, ESTIMATEScore, and TumorPurity in the COAD cohort. (c) Correlation between RIG‐I and 28 regulated immune cells across all the seven cohorts. The color indicates the correlation coefficient. The asterisks indicate a statistically significant *p*‐value calculated using Spearman correlation analysis. (d) Heatmap showed the difference in 28 types of immune cells between high‐ and low‐RIG‐I groups of the COAD cohort. (e) Bar graph showed the |log2‐fold change (FC)| of the 28 regulated immune cells infiltrating high versus low RIG‐I‐expressing CRC samples. (f) Gene set enrichment analysis (GSEA) showed the significant functional gene sets enriched in COAD with RIG‐I highly expressed. *p < 0.05, **p < 0.01, ***p < 0.001, and ****p < 0.0001 versus the control group. COAD, colon adenocarcinoma; CRC, colorectal cancer.

Based on the strong correlation between MSI and immunotherapy, along with reports on RIG‐I expression and immunity in other tumors [10, 19], we first calculated immune scores, stromal scores, estimate scores, and tumor purity. We found that the high RIG‐I expression group had significantly higher stromal and immune scores compared to the low RIG‐I expression group across the seven cohorts (Figures 3a and S5). Additionally, RIG‐I expression was highly positively correlated with these scores (Figures 3b and S5). In contrast, tumor purity was lower in the high RIG‐I expression group, suggesting an increased presence of immune cells in these tumors (Figures 3a and S5). Furthermore, we applied the ssGSEA algorithm to assess the infiltration levels of 28 immune cell types in each CRC sample. We observed that RIG‐I was positively correlated with most immune cells across the seven cohorts (Figure 3c,d). These results indicated that tumors in the high RIG‐I group had a higher enrichment of antitumor immune cells, including effector memory CD8+ T cells, natural killer cells, and type 1 T helper cells (Figures 3e and S6). To further explore the potential biological functions of RIG‐I in CRC, we conducted gene set enrichment analysis (GSEA), which revealed several significantly enriched immune‐related pathways. High RIG‐I expression was notably associated with enrichment in the “interferon alpha (IFN‐α) response” and “interferon gamma response” pathways across all seven cohorts (Figures 3f and S7). Together, these findings suggest that high RIG‐I expression is linked to the activation of antitumor immune cells and the interferon response in CRC.

### 3.4 Upregulation of RIG‐I Promotes IFN‐α‐Induced Cell Death by Inducing Phosphorylation of STAT1 in CRC

**FIGURE 4:**
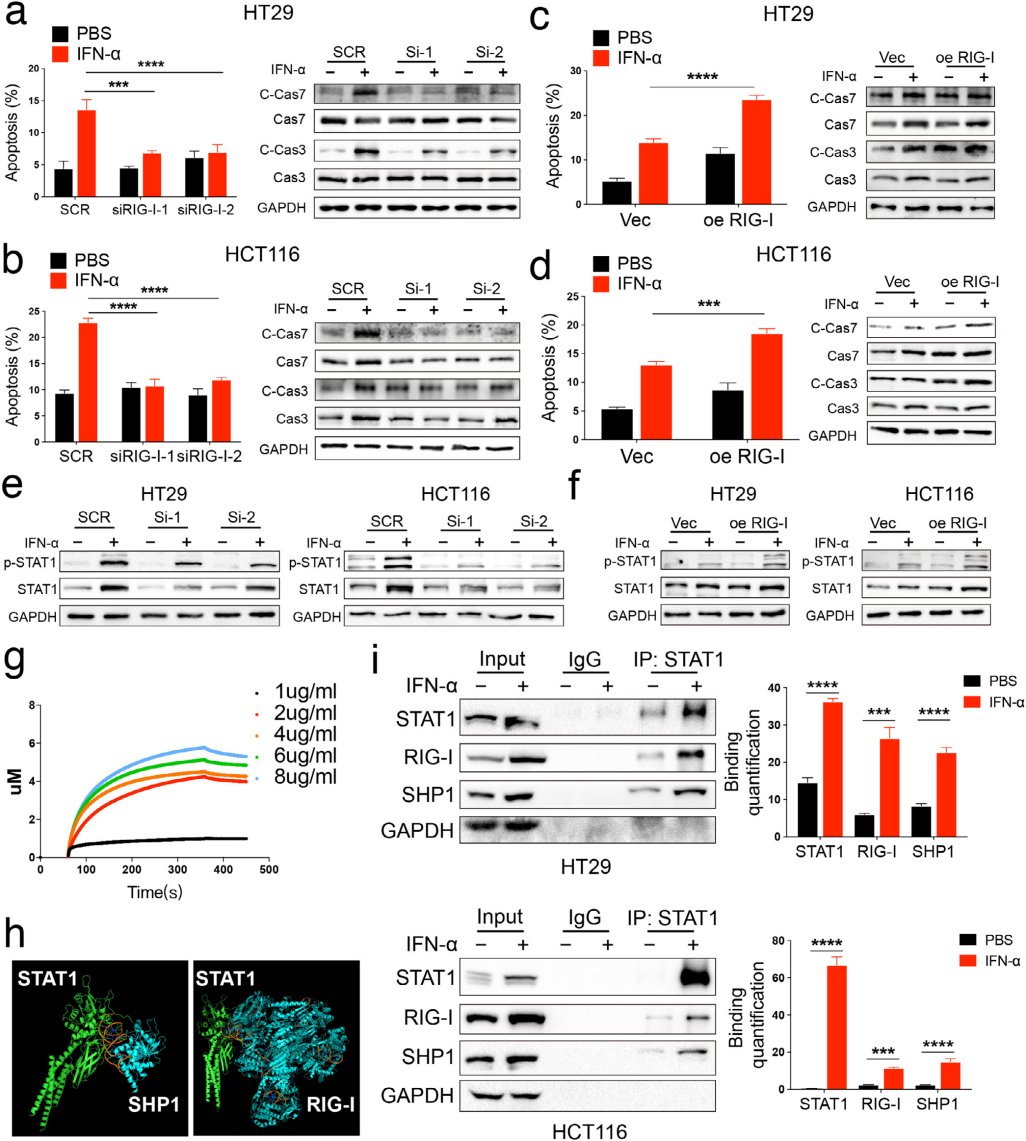
RIG‐I promotes interferon‐α (IFN‐α)‐induced apoptosis by inducing phosphorylation of STAT1 in CRC. (a‐b) HT29 and HCT116 cells were transfected with scramble and RIG‐I small interfering RNA (siRNA) for 48 h, and then treated with 2000 U/mL human recombinant IFN‐α for 48 h. Cells were isolated, stained with annexin V and PI, and analyzed by flow cytometry. Also, cell lysates were collected and analyzed by western blot. (c‐d) HT29 and HCT116 cells were transfected with Vec and RIG‐I overexpression plasmids for 48 h and then treated with 2000 U/mL human recombinant IFN‐α for 48 h. Cells were isolated, stained with annexin V and PI, and analyzed by flow cytometry. Also, cell lysates were collected and analyzed by western blot. (e) HT29 and HCT116 cells were transfected with scramble and RIG‐I siRNA for 48 h, and then treated with 2000 U/mL human recombinant IFN‐α for 48 h, and cell lysates were collected and analyzed by western blot. (f) HT29 and HCT116 cells were transfected with Vec and RIG‐I overexpression plasmids for 48 h and then treated with 2000 U/mL human recombinant IFN‐α for 48 h. Cell lysates were collected and analyzed by western blot. (g) Kinetic interactions of RIG‐I and the STAT1 protein were determined by Biolayer interferometry. (h) Prediction of the STAT1 and SHP1 (left) and STAT1 and RIG‐I (right) interactions using Cluspro (https://cluspro.bu.edu/queue.php) and shown by PyMOL. (i) HT29 cells and HCT 116 cells were treated with 2000 U/mL human IFN‐α for 48 h. Then, cell lysates were used for immunoprecipitation with anti‐STAT1 antibody and analyzed by western blot with the indicated antibodies. *p < 0.05, **p < 0.01, ***p < 0.001, and ****p < 0.0001 versus the control group. The Cancer Genome Atlas; COAD, colon adenocarcinoma; CRC, colorectal cancer; READ, rectum adenocarcinoma; SCR, scramble; Vec, vector.

We hypothesized that RIG‐I plays a key role in mediating IFN‐α resistance in CRC. To test this hypothesis, we silenced RIG‐I expression in HT29 and HCT116 cells using small interfering RNA (siRNA) (Figure S8a,c). Additionally, we overexpressed RIG‐I in these cells with plasmids (Figure S8b,d). As shown in our previous studies, IFN‐α treatment primarily induces apoptosis and slight cell cycle arrest [14]. We then assessed the effect of RIG‐I on IFN‐α‐induced cell death in CRC cells. We observed significantly less cell death in the si‐RIG‐I group compared to control CRC cells under IFN‐α treatment (Figures 4a,b and S9a,c). Conversely, more cell death occurred in the RIG‐I overexpression group compared to control cells under IFN‐α stimulation (Figure 4c,d and S9b,d).

Next, we investigated the underlying mechanism of RIG‐I‐mediated IFN‐α‐induced cell death. As previously reported, phosphorylation of signal transducer and activator of transcription 1 (STAT1) is activated during IFN‐α treatment [26]. In our study, phosphorylation of STAT1 was reduced in RIG‐I‐knockdown cells compared to controls after IFN‐α stimulation (Figure 4e). In contrast, RIG‐I overexpression significantly enhanced STAT1 phosphorylation (Figure 4f). To further confirm the relationship between RIG‐I and STAT1, we conducted a biolayer interferometry (BLI) analysis, which showed that STAT1 directly binds to RIG‐I in vitro (Figure 4g). Additionally, our previous research indicated that SHP1 competes with RIG‐I for binding to STAT1, thereby inhibiting STAT1 phosphorylation in melanoma [14]. To further explore this, we used ClusPro to dock SHP1 (4GRY), RIG‐I (6KYV), and p‐STAT1 (1BF5) proteins [27]. The docking results revealed that SHP1 and RIG‐I share similar binding sites with STAT1 (Figure 4h). We validated this prediction using immunoprecipitation and found that the binding of SHP1 and RIG‐I to STAT1 was significantly increased in the IFN‐α treatment group (Figure 4i). Thus, RIG‐I promotes STAT1 phosphorylation by competing with SHP1 for STAT1 binding.

### 3.5 RIG‐I Promotes CXCL10 and CXCL11 Expression Under IFN‐α Stimulation in CRC

**FIGURE 5:**
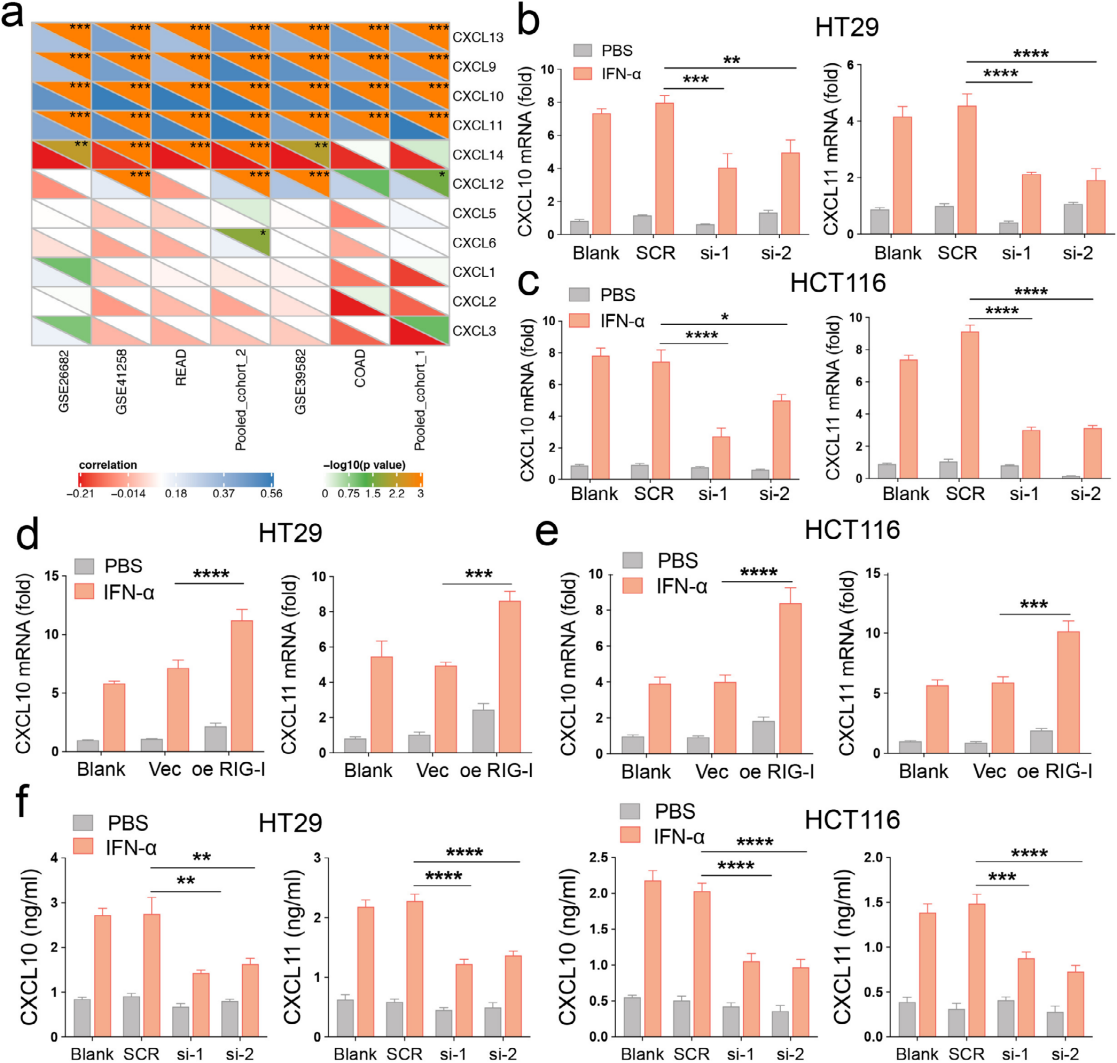
RIG‐I promotes CXCL10 and CXCL11 expression under the interferon‐α (IFN‐α) treatment in CRC. (a) Correlation between RIG‐I and CXCL genes across all the seven cohorts. The color indicates the correlation coefficient. The asterisks indicate a statistically significant *p*‐value calculated using Spearman correlation analysis. (b‐c) HT29 and HCT116 cells were transfected with scramble and RIG‐I siRNA for 48 h and then treated with 2000 U/mL human recombinant IFN‐α for 48 h. The CXCL10 and CXCL11 expression were analyzed by RT‐qPCR. (d‐e) HT29 and HCT116 cells were transfected with scramble and RIG‐I siRNA for 48 h and then treated with 2000 U/mL human recombinant IFN‐α for 48 h. The CXCL10 and CXCL11 expression were analyzed by RT‐qPCR. (f) Culture supernatants of HT29 and HCT116 cells were collected after 48 h IFN‐α treatment, and the protein levels of CXCL10 and CXCL11 were measured using ELISA. *p < 0.05, **p < 0.01, ***p < 0.001, and ****p < 0.0001 versus the control group. The Cancer Genome Atlas; COAD, colon adenocarcinoma; CRC, colorectal cancer; SCR, scramble; Vec, vector.

Given that patients with high RIG‐I expression are more responsive to IFN‐α, we analyzed the correlation between RIG‐I expression and chemokine family members. Chemokines play a crucial role in both innate and acquired immune responses [28]. Our results showed that RIG‐I expression was significantly positively correlated with CXCL9, CXCL10, CXCL11, and CXCL13 levels across all seven cohorts (Figure 5a). To explore this further, we examined the expression of these chemokines following IFN‐α treatment. The results indicated that IFN‐α primarily induced CXCL10 and CXCL11 expression in HT29 and HCT116 cells (Figure S10). Next, we focused on the relationship between CXCL10/11 and RIG‐I. We found that the mRNA levels of CXCL10 and CXCL11 were reduced in RIG‐I‐knockdown cells but elevated in RIG‐I‐overexpressing cells following IFN‐α treatment (Figure 5b–e). ELISA results confirmed these changes at the protein level (Figure 5f). The canonical RIG‐I signaling pathway involves mitochondrial antiviral signaling protein (MAVS)‐dependent activation of interferon regulatory factors 3 and 7 (IRF3/7), which leads to type I IFN release and STAT1 activation [29]. To investigate the mechanism, we first silenced MAVS, IRF3, or IRF7 in RIG‐I‐overexpressing cells. CXCL10 and CXCL11 expression was partially restored in these cells compared to the knockdown group alone (Figure S11). Given the sensitivity of RIG‐I recognition, residual MAVS activity may still explain the impact of RIG‐I overexpression in these cells. To further validate this, we generated MAVS knockout (KO) MC38 cell lines (Figure S12a,b). In MAVS KO MC38 cells, RIG‐I overexpression resulted in higher mRNA levels of CXCL10 and CXCL11 under IFN‐α treatment compared to the knockout groups alone (Figure S12c). In summary, our findings suggest that RIG‐I enhances the CRC response to IFN‐α by promoting the expression and secretion of CXCL10 and CXCL11.

### 3.6 RIG‐I is Essential for Responsiveness to IFN‐α Plus Checkpoint Blockade in CRC

**FIGURE 6:**
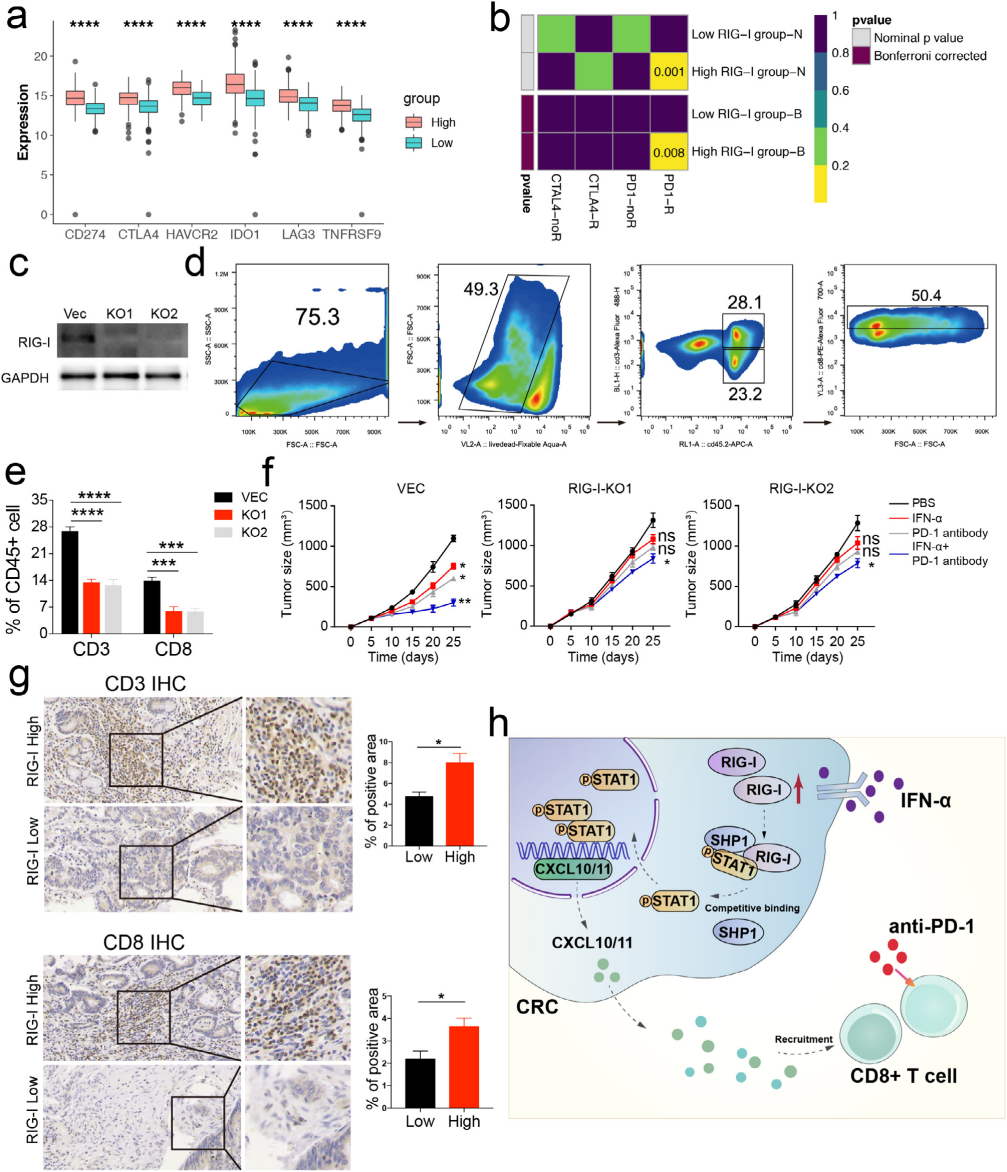
High expression of RIG‐I is essential for responsiveness to IFN‐α plus checkpoint blockade in CRC. (a) The expression differences of immune checkpoint genes, including LAG3, CD274, IDO1, HAVCR2, CTLA4, and TNFRSF9 in COAD with high versus low RIG‐I expression, were separated by the median expression of RIG‐I. (b) High RIG‐I expression may be more sensitive to the PD‐1 inhibitor by submap analysis in the COAD cohort. (c) Transfection efficiency of two RIG‐I knockout MC38 cells. (d) Representative dot blot of flow cytometric analysis of CD45.2+ CD3+ CD8+ cells. (e) The quantitative percentage of CD3 and CD8 between Vec and RIG‐I knockout tumors was shown. (f) MC38 cells (1 × 10^6^/ml) were subcutaneously injected into the right flanks of mice. Seven days later, the mice were randomly divided into control, IFN‐α, anti‐PD1, and IFN‐α + anti‐PD‐1 groups. Intratumoral injections of anti‐PD‐1 (250 μg per mouse) or IFN‐α (2 × 10^5^ U/mouse) were performed twice per day until the end of Day 25. The control groups received the same doses of IgG or PBS in the same way at the same time. Tumor volumes were measured every 5 days. (g) Representative immunohistochemical staining of CD3 and CD8 in RIG‐I high and low expression CRC samples. The corresponding quantitative results were also shown. (h) A schematic shows the RIG‐I‐mediated IFN‐α plus anti‐PD‐1 signaling in CRC cells. *p < 0.05, **p < 0.01, ***p < 0.001, and ****p < 0.0001 versus the control group. COAD, colon adenocarcinoma; CRC, colorectal cancer.

Recent studies have highlighted the importance of IFN signaling in driving immune checkpoint blockade [30, 31, 32]. Therefore, we explored the correlation between RIG‐I expression and key immune checkpoints, including PD‐L1, CTLA‐4, LAG‐3, HAVCR2, IDO1, and TNFRSF9. We found that immune checkpoint expression was upregulated in the high RIG‐I group (Figures 6a, and S13). Next, we assessed the potential response to immunotherapy by comparing our RIG‐I expression profiles with a published melanoma immunotherapy dataset [33]. We found that patients with high RIG‐I expression across different cohorts were more likely to respond to anti‐PD‐1 therapy (Figures 6b and S14). These results suggest that RIG‐I expression could predict the efficacy of immunotherapy in CRC.

However, these findings were based on bioinformatic analysis, so we further validated our results. We generated RIG‐I knockout (KO) MC38 cells (Figure 6c) and found that these cells had lower CXCL10 and CXCL11 expression, both at the mRNA and protein levels, following IFN‐α treatment (Figure S15). These cells were then used to establish tumor‐bearing mouse models. Flow cytometry analysis revealed lower levels of CD3+ CD8+ T cells in the RIG‐I KO MC38 group compared to the Vec cells (Figure 6d,e).

Given that combination therapies have been shown to enhance the efficacy of checkpoint blockade, including IFN‐α [23], we administered IFN‐α plus anti‐PD‐1 therapy to the Vec MC38 and RIG‐I KO MC38 mouse models. The results indicated that the combination therapy was less effective in the RIG‐I KO group, with IFN‐α or PD‐1 antibody alone failing to inhibit tumor growth (Figure 6f). Furthermore, patients with high RIG‐I expression had higher infiltration of CD3+ CD8+ T cells compared to those with low RIG‐I expression (Figure 6g). In conclusion, our data suggest that RIG‐I expression is essential for the effectiveness of IFN‐α and anti‐PD‐1 combination therapy, offering potential clinical benefits in CRC treatment (Figure 6h).

## 4 Discussion

In this study, we identified RIG‐I as a tumor suppressor in CRC using public databases, human CRC samples, and cell lines. Through bioinformatic analyses, we confirmed the association between RIG‐I and the immune microenvironment, including MSI, immune cell composition, chemokines, and immune checkpoints. Mechanistically, RIG‐I promoted STAT1 phosphorylation by competing with SHP1, which enhanced IFN‐α‐induced apoptosis in CRC cells. Additionally, RIG‐I increased the expression of CXCL10/11, boosting the response to IFN‐α therapy. Notably, patients with higher RIG‐I expression were predicted to have a better response to anti‐PD‐1 blockade. Tumor‐bearing mouse experiments further validated that combining IFN‐α with anti‐PD‐1 therapy in the presence of RIG‐I could provide additional therapeutic benefits.

The molecular characteristics of CRC and biomarkers for its diagnosis and prognosis have garnered significant attention [34]. RIG‐I has been identified as a tumor suppressor in various cancers, including HCC, melanoma, and acute lymphoblastic leukemia [14, 19, 35]. In this study, involving 2246 CRC patients, we found that RIG‐I expression was significantly reduced in CRC, supporting previous findings by Zhu et al., who reported downregulation of RIG‐I in colitis‐associated CRC [36]. Our results contribute to the growing evidence of RIG‐I expression differences in CRC. However, we also found that RIG‐I expression did not correlate with CRC prognosis or clinical features, such as TNM stage or pathological grade. This contrasts with other cancers, where RIG‐I expression has been linked to better survival (HCC) or poorer outcomes (ovarian cancer) [19, 37]. Therefore, further research is needed to explore the relationship between RIG‐I and CRC clinical characteristics. Mechanistically, previous studies have shown that RIG‐I knockout disrupts gut microbiota by inhibiting IgA and IL6‐STAT3‐dependent Reg3γ expression [36], while others have reported that RIG‐I regulates biomarkers like polybromo‐1 [38] and insulin‐like growth factor‐1 receptor [39] in CRC. Despite this, the role of RIG‐I in CRC immunotherapy remains unexplored. Therefore, elucidating the relationship between RIG‐I expression levels and the onset, progression, and response to immunotherapy in colorectal cancer will significantly enhance personalized treatment strategies for patients.

Given the significant genetic, epigenetic, and clinical heterogeneity of tumors, we tried to investigate the potential biological relationship between RIG‐I and factors such as MSI, genetic mutations, and clinical phenotypes. The MSI subtype was characterized by high levels of immune cell infiltration, mutation burden, and immune checkpoint expression [40]. However, the relationship between the occurrence and development of MSI and RIG‐I is still unclear. Here, our results were further validated by this result through a larger sample size and pan‐cancer analysis. Moreover, patients with high RIG‐I expression also showed a higher mutation rate, especially the BRAF V600E mutation. This could be supported by the observation that the BRAF V600E mutation was more likely to occur in MSI CRC than in MSS CRC (40% vs. 7%) [41]. In addition, we found that proximal CRC showed higher RIG‐I expression than distal CRC. This was consistent with the findings of Minoo et al. [42]. They found that the frequency of BRAF V600E mutation and the MSI‐H phenotype was significantly increased in proximal CRC. Therefore, we postulate that RIG‐I may affect CRC progression primarily by regulating the immune response. Furthermore, the mechanisms underlying the regulation of RIG‐I in relation to MSI and gene mutations still require further biological research. Such studies will offer important theoretical insights into the role of genetics in tumor development and treatment responses.

To further explore the critical role of RIG‐I, we divided CRC patients into two groups based on median RIG‐I expression. Our results showed that most immune cells in the high RIG‐I expression group were activated, with a predominance of antitumor immune cells. This finding supports RIG‐I as a potential novel immunotherapy biomarker for CRC. GSEA revealed that the most relevant function was the IFN‐α response, consistent with RIG‐I's known role as an ISG. Previous studies have shown significant upregulation of RIG‐I expression following IFN‐α treatment [19], yet its specific role in CRC remained unclear. To address this, we treated two CRC cell lines with IFN‐α and observed that RIG‐I overexpression enhanced IFN‐α‐induced cell death by activating STAT1 phosphorylation. This suggests that RIG‐I could improve the response to IFN‐α therapy in CRC. Mechanistically, we found that RIG‐I promoted STAT1 phosphorylation by competing with SHP1 for binding to STAT1. Notably, STAT1 has been identified as a favorable prognostic biomarker in CRC [43, 44], which may help explain how RIG‐I exerts its antitumor effects. Interestingly, RIG‐I expression alone had no significant effect on CRC cell death, which partly explains its lack of correlation with clinicopathological features. Thus, RIG‐I induced by IFN‐α appears to amplify IFN‐α‐induced cell death in CRC.

Immune checkpoint inhibitors (ICIs) have shown promising therapeutic responses in patients with various types of refractory cancer [45, 46]. In CRC, however, only patients with the MSI‐H/dMMR subtype respond to ICI therapy, particularly anti‐PD‐1 or anti‐PD‐L1 drugs [47, 48]. Interestingly, the expression of several immune checkpoints was found to be elevated in the high RIG‐I expression group, with PD‐L1 showing the strongest positive correlation with RIG‐I expression. This suggests that IFN signaling plays a critical role in promoting responses to ICI therapy [49]. Additionally, increased expression or secretion of CXCL10/11 has been shown to enhance tumor response to ICI immunotherapy [50]. Our data indicated that IFN‐α stimulation significantly boosted CXCL10/11 expression in RIG‐I overexpressing cells. Given that RIG‐I, PD‐L1, and CXCL10/11 are all ISGs, their correlated expression pattern is expected. Importantly, even when MAVS/IRF3/IRF7 signaling was inhibited in RIG‐I overexpressing cells, CXCL10 and CXCL11 expression could not be fully suppressed. Furthermore, the increased CXCL10/11 expression likely contributes to enhanced recruitment of activated CD8+ T cells into the tumor [51]. In vivo, we confirmed that RIG‐I knockout significantly reduced CD3+ CD8+ T cell infiltration in tumor tissues, while high RIG‐I expression correlated with increased CD8+ T cell infiltration. While Heidegger et al. previously proposed that RIG‐I activation was critical for responsiveness to checkpoint blockade, their results—primarily derived from melanoma B16 cell lines—showed that RIG‐I activation enhanced the therapeutic effects of anti‐CTLA4 therapy [52]. Their use of the MSS CRC cell line CT26 yielded similar results, although they found no significant benefit from RIG‐I activation in improving anti‐PD‐1 therapy. In contrast, our study demonstrated that combined anti‐PD‐1 and IFN‐α treatment reduced tumor volumes, an effect that was abolished in mice bearing RIG‐I knockout MC38 cells. Additionally, RIG‐I not only enhances the activity of antitumor immune cells but also suppresses the activity of immunosuppressive cells. Fournier et al. reported that RIG‐I activation can inhibit the immunosuppressive activity of Treg cells [53]. Furthermore, activating RIG‐I has been shown to enhance the ability of Toll‐like receptor agonists to suppress the activity of myeloid‐derived suppressor cells [54]. This helps explain why, in our results, colorectal cancer tissues with high RIG‐I expression exhibit immune cell infiltration with varied immune activities, ultimately leading to an antitumor effect. Therefore, our study may provide new ideas for improving the response rates to anti‐PD‐1 therapy in CRC patients with high RIG‐I expression. Importantly, a recent study reported novel polymeric carriers to enhance the delivery and activity of 3pRNA, a RIG‐I agonist, which might provide a novel approach for combination therapy with ICIs in the future [18, 55].

Based on the above results, we determined that RIG‐I plays an important role in the antitumor immunity of CRC. Meanwhile, as an ISG, the activation of RIG‐I can further upregulate the expression of the downstream molecules, MAVS/IRF3/IRF7, thereby promoting interferon expression and secretion. In turn, interferon secretion further activates RIG‐I, forming a large number of positive feedback signals, which promote the secretion of chemokines. Finally, patients with high RIG‐I expression had increased CD8+ T cell infiltration compared with patients with low RIG‐I expression. However, some limitations in our study require further elucidation. First, due to the low proportion of MSI CRC, we did not collect enough samples with a definite diagnosis of MSI to confirm our results. Second, the regulatory mechanisms of RIG‐I in MSI or MSS CRC are still unknown. Third, since IFN‐α and anti‐PD‐1 combination therapy have not yet been tested in humans, its potential unintended side effects remain uncertain. Furthermore, this study provides several recommendations for future research. First, exploring the role of RIG‐I in colorectal cancer from a genetics perspective could help expand the scope of immunotherapy research. Second, developing efficient and accessible detection methods for measuring RIG‐I expression in colorectal cancer patients would support clinical studies evaluating the effectiveness of IFN‐α and anti‐PD‐1 combination therapy in patients with high RIG‐I expression.

In conclusion, our data clearly indicate that upregulation of RIG‐I is critical for responsiveness to IFN‐α plus anti‐PD‐1 therapy in CRC. Our study may provide a novel concept for combinatorial ICI immunotherapy for CRC.

## Author Contributions

**Haihang Nie:** writing – original draft, methodology. **Shilin Fang:** writing – review and editing, visualization. **Rui Zhou:** formal analysis, writing – review and editing. **Yifan Jia:** project administration. **Jingkai Zhou:** methodology, validation. **Yumei Ning:** supervision, formal analysis. **Yali Yu:** software, data curation. **Yuntian Hong:** visualization, investigation. **Fei Xu:** formal analysis, investigation. **Qiu Zhao:** project administration, funding acquisition. **Jiayan Nie:** funding acquisition, supervision, resources. **Fan Wang:** project administration, funding acquisition, writing – review and editing, resources.

## Ethics Statement

The protocol for collecting human biospecimens in this study was approved by the Research Ethics Committee of Zhongnan Hospital of Wuhan University, and written informed consent was obtained from all patients (Grant No. 2024049). All the methods were carried out in accordance with the relevant guidelines under the ethical approval and consent to participate section. The study protocol, including animal experiments, was approved by the Ethics Committee of Zhongnan Hospital of Wuhan University (SQ20200208).

## Conflicts of Interest

The authors declare no conflicts of interest.

## Supporting information


**Figure S1.** PCA showed the batch effect of pooled cohorts 1 and 2 before and after the combination.
**Figure S2:** Survival analysis of RIG‐I based on the (a) COAD, (b) READ, (c, d) GSE39582, and (e) tissue microarray.
**Figure S3:** Correlation of RIG‐I expression with clinical phenotypes in GSE39582, READ, GSE26682 and GSE41258 cohorts.
**Figure S4:** Comparison of RIG‐I expression between BRAF V600E, KRAS, TP53, and Pi3kca mutations in GSE39582, GSE41258, and pooled cohort 2 cohorts.
**Figure S5:** The boxplot indicated the differences in immune score, stromal score, estimate score, and tumor purity in six cohorts with high versus low RIG‐I expression separated by median expression of RIG‐I. The heatmap indicated the correlation between RIG‐I expression and immune score, stromal score, estimate score, and tumor purity in six cohorts with high versus low RIG‐I expression separated by median expression of RIG‐I.
**Figure S6:** Bubble plot representation showing the log2 fold change of immune cell infiltration in seven cohorts with high versus low RIG‐I expression separated by median expression of RIG‐I.
**Figure S7:** Gene set enrichment analysis (GSEA) showed the significant functional gene sets enriched in Pooled cohort 1, Pooled cohort 2, READ, GSE39582, GSE26682, and GSE41258 cohorts with RIG‐I highly expressed.
**Figure S8:** Transfection efficiency of RIG‐I plasmid and siRNA in CRC. Real‐time PCR (left) and western blotting (right) were used to analyze the mRNA and protein expression levels of RIG‐I in HT29 cells (a) and HCT116 cells (c) transfected with SCR and siRNA (si‐1, si‐2). Real‐time PCR (left) and western blotting (right) were used to analyze the mRNA and protein expression levels of RIG‐I in HT29 cells (b) and HCT116 cells (d) transfected with Vec and RIG‐I plasmids (oe RIG‐I). *p < 0.05, **p < 0.01, ***p < 0.001, and ****p < 0.0001 versus control group. SCR, scramble; Vec, vector; oe, overexpression.
**Figure S9:** HT29 and HCT116 cells were transfected with scramble and RIG‐I small interfering RNA (siRNA) for 48 h, and then treated with 2000 U/mL human recombinant IFN‐α for 48 h. Cells were isolated, stained with annexin V and PI, and analyzed by flow cytometry. Quadrants from the lower left (counterclockwise) represent healthy, early apoptotic, late apoptotic, and necrotic cells, respectively. The only Annexin V–positive cells were regarded as apoptotic cells.
**Figure S10:** The difference expression levels of CXCL9, 10, 11, and 13 under the interferon‐α treatment in HT29 (a) and HCT116 cells (b) by PCR, respectively.
**Figure S11:** HT29 (a) and HCT116 cells (b) were transfected with RIG‐I overexpression plasmid, mitochondrial antiviral signaling protein (MAVS) siRNA, dependent interferon regulatory factor 3/7 (IRF3/7) siRNA for 48 h, and then treated with 2000 U/mL human recombinant IFN‐α for 48 h. The CXCL10 and CXCL11 expression were analyzed by RT‐qPCR. *p < 0.05, **p < 0.01, ***p < 0.001, ****p < 0.0001 versus control group.
**Figure S12:** (a) Top, a related portion of MAVS genomic structure. Bottom, sequences of the targeted region and the two knockout alleles (KO‐1 and KO‐2). Two guide RNAs (gRNAs) were used to achieve the targeting. (b) Transfection efficiency of two MAVS KO MC38 cells. (c) MAVS KO MC38 cells were transfected with RIG‐I overexpression plasmid for 48 h and then treated with 2000 U/mL human recombinant IFN‐α for 48 h. The CXCL10 and CXCL11 expression were analyzed by RT‐qPCR. KO, knockout. ****p < 0.0001 versus control group.
**Figure S13:** The expression differences in immune checkpoint genes, including LAG3, CD274, IDO1, HAVCR2, CTLA4, and TNFRSF9 in six cohorts with high versus low RIG‐I expression separated by median expression of RIG‐I.
**Figure S14:** High RIG‐I expression may be more sensitive to the PD‐1 inhibitor by SubMap analysis in pooled cohort 1, pooled cohort 2, READ, and GSE39582 cohorts. *p < 0.05, **p < 0.01, ***p < 0.001, and ****p < 0.0001 versus control group.
**Figure S15:** (a) MC38 Vec and RIG‐I knockout (KO) cells were treated with 200 ng/mL murine recombinant IFN‐α for 48 h. The CXCL10 and CXCL11 expression were analyzed by RT‐ qPCR. (b) Culture supernatants were collected after 48 h treatment, and the protein levels of CXCL10 and CXCL11 were measured using ELISA. **p < 0.01 and ****p < 0.0001 versus control group.

**Table S1.** Clinical cohorts used in this study.

**Table S2.** The detailed characteristics of patients in all cohorts used in this study.

**Table S3.** The list of genes in 28 immune cells.

**Table S4.** Sequences of primers for PCR.

**Table S5.** Sequences of primers for CRISPR.

Data S1.

## Data Availability

The data that support the findings of this study are available from the corresponding author upon reasonable request.
