# Supplementary material for: Upregulation of RIG‐I is Critical for Responsiveness to IFN‐α Plus Anti‐PD‐1 in Colorectal Cancer
Source: Cancer Med. 2025 Mar 21;14(6):e70802. doi: 10.1002/cam4.70802 (PMC11926914; doi:10.1002/cam4.70802)
Supplement: Supplementary file 1 — Figure S1. PCA showed the batch effect of pooled cohorts 1 and 2 before and after the combination. Figure S2: Survival analysis of RIG‐I based on the (a) COAD, (b) READ, (c, d) GSE39582, and (e) tissue microarray. Figure S3: Correlation of RIG‐I expression with clinical phenotypes in GSE39582, READ, GSE26682 and GSE41258 cohorts. Figure S4: Comparison of RIG‐I expression between BRAF V600E, KRAS, TP53, and Pi3kca mutations in GSE39582, GSE41258, and pooled cohort 2 cohorts. Figure S5: The boxplot indicated the differences in immune score, stromal score, estimate score, and tumor purity in six cohorts with high versus low RIG‐I expression separated by median expression of RIG‐I. The heatmap indicated the correlation between RIG‐I expression and immune score, stromal score, estimate score, and tumor purity in six cohorts with high versus low RIG‐I expression separated by median expression of RIG‐I. Figure S6: Bubble plot representation showing the log2 fold change of immune cell infiltration in seven cohorts with high versus low RIG‐I expression separated by median expression of RIG‐I. Figure S7: Gene set enrichment analysis (GSEA) showed the significant functional gene sets enriched in Pooled cohort 1, Pooled cohort 2, READ, GSE39582, GSE26682, and GSE41258 cohorts with RIG‐I highly expressed. Figure S8: Transfection efficiency of RIG‐I plasmid and siRNA in CRC. Real‐time PCR (left) and western blotting (right) were used to analyze the mRNA and protein expression levels of RIG‐I in HT29 cells (a) and HCT116 cells (c) transfected with SCR and siRNA (si‐1, si‐2). Real‐time PCR (left) and western blotting (right) were used to analyze the mRNA and protein expression levels of RIG‐I in HT29 cells (b) and HCT116 cells (d) transfected with Vec and RIG‐I plasmids (oe RIG‐I). *p < 0.05, **p < 0.01, ***p < 0.001, and ****p < 0.0001 versus control group. SCR, scramble; Vec, vector; oe, overexpression. Figure S9: HT29 and HCT116 cells were transfected with scramble and RIG‐I sma [file CAM4-14-e70802-s001.doc]

**
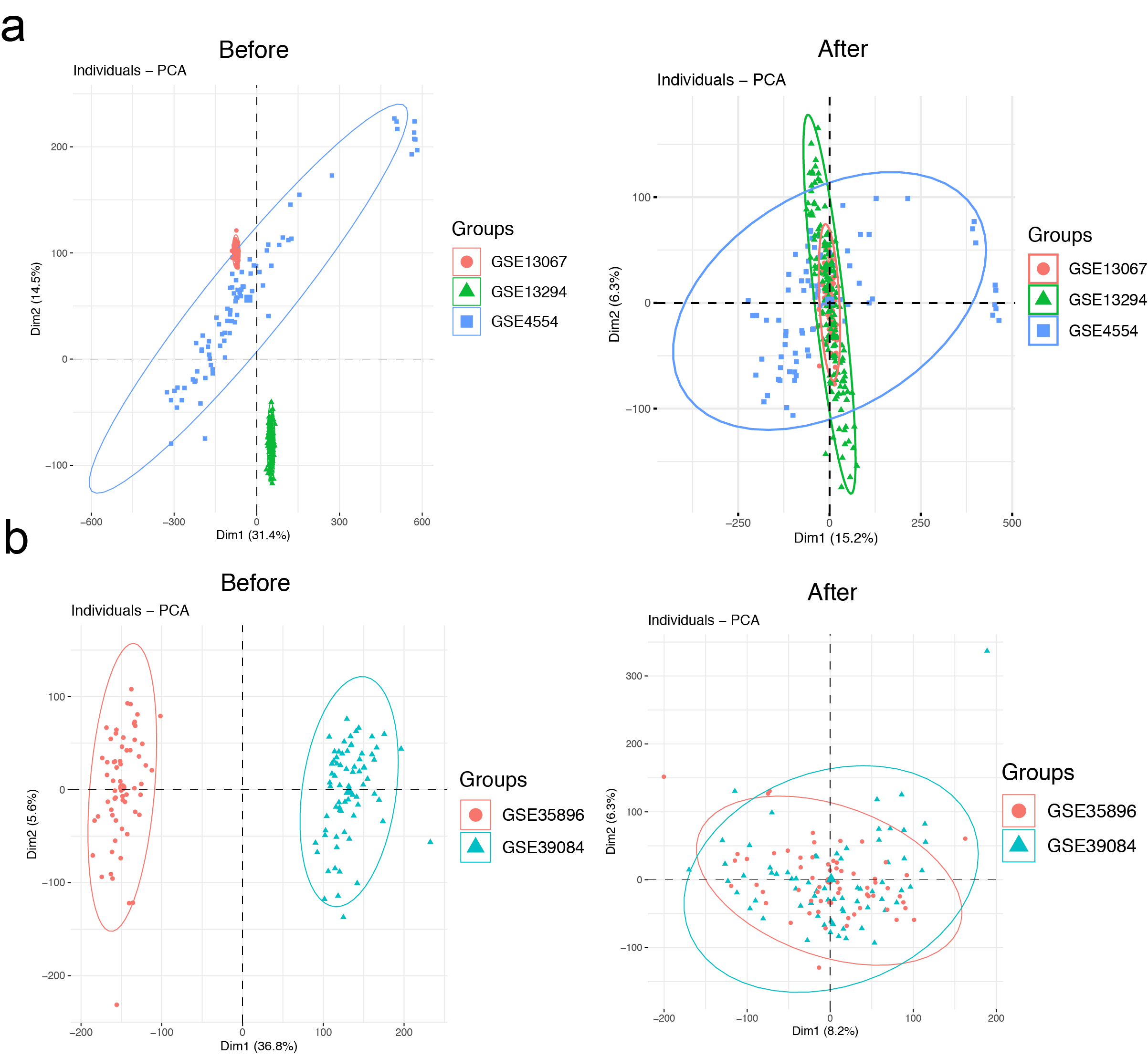
**

**Fig. S1:** PCA showed the batch effect of pooled cohort 1 and 2 before and after the combination.


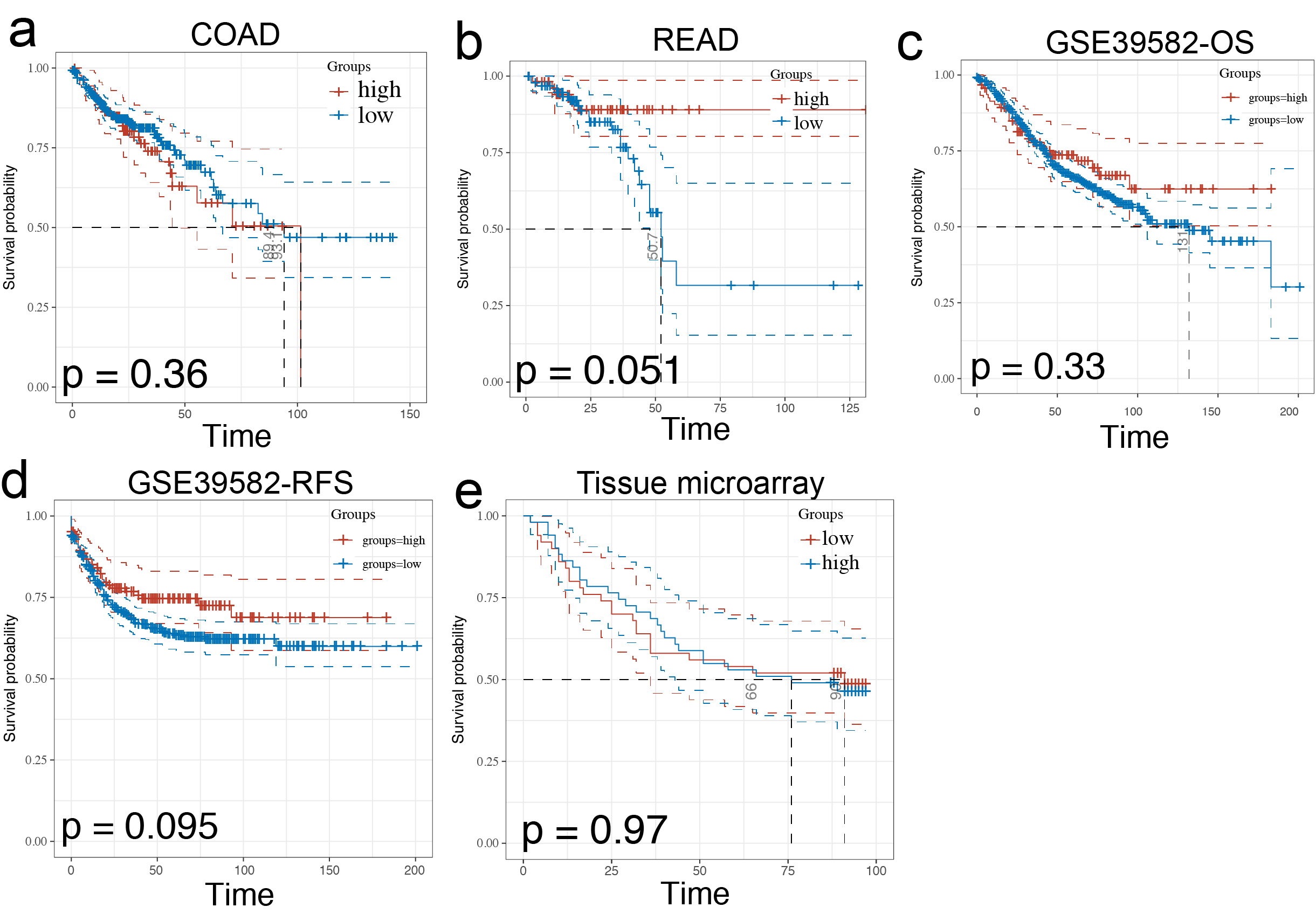


**Fig. S2:** Survival analysis of RIG-I based on the (a) COAD, (b) READ, (c, d) GSE39582 and (e) tissue microarray.


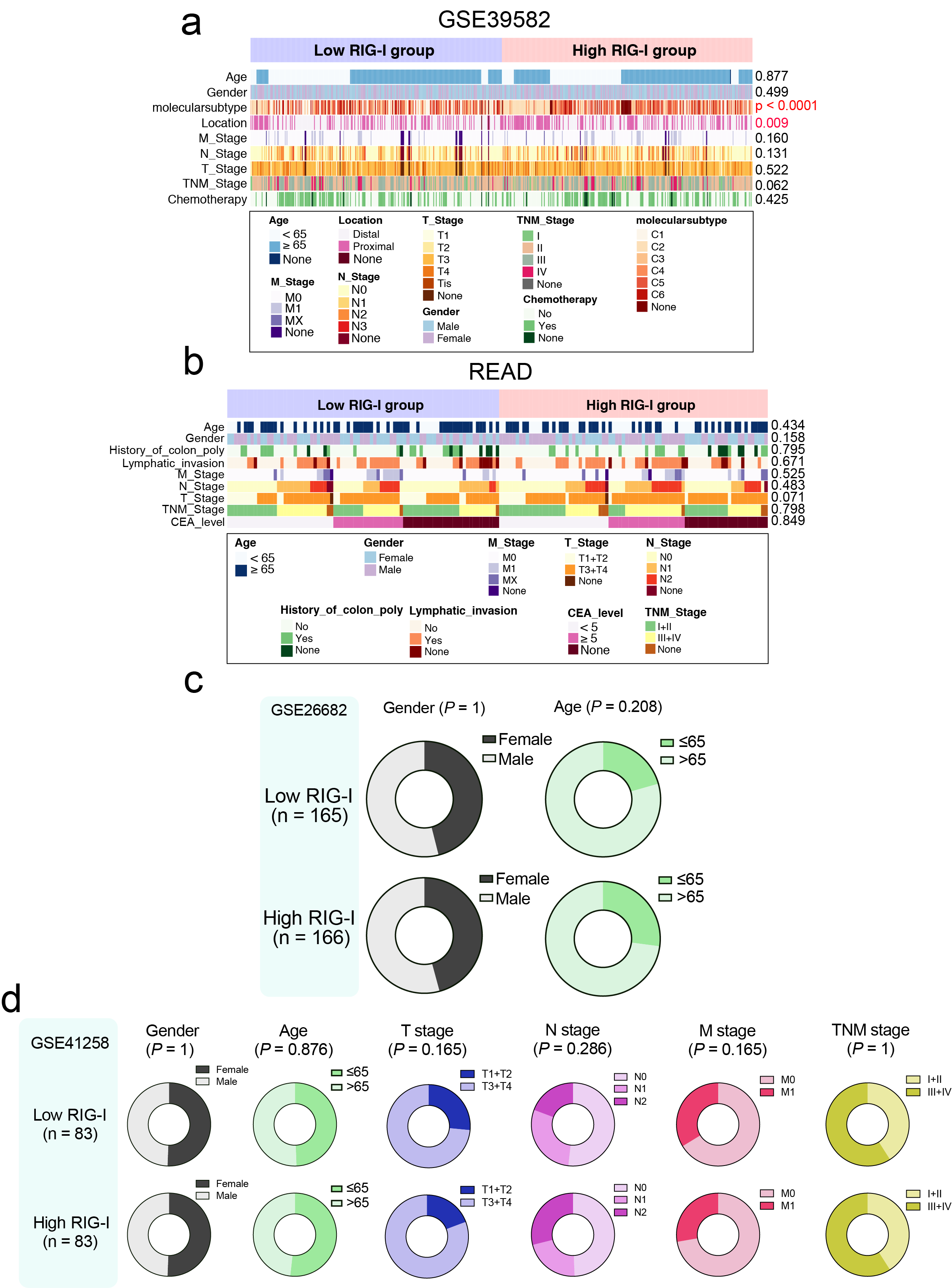


**Fig. S3:** Correlation of RIG-I expression with clinical phenotypes in GSE39582, READ, GSE26682 and GSE41258 cohorts.

**
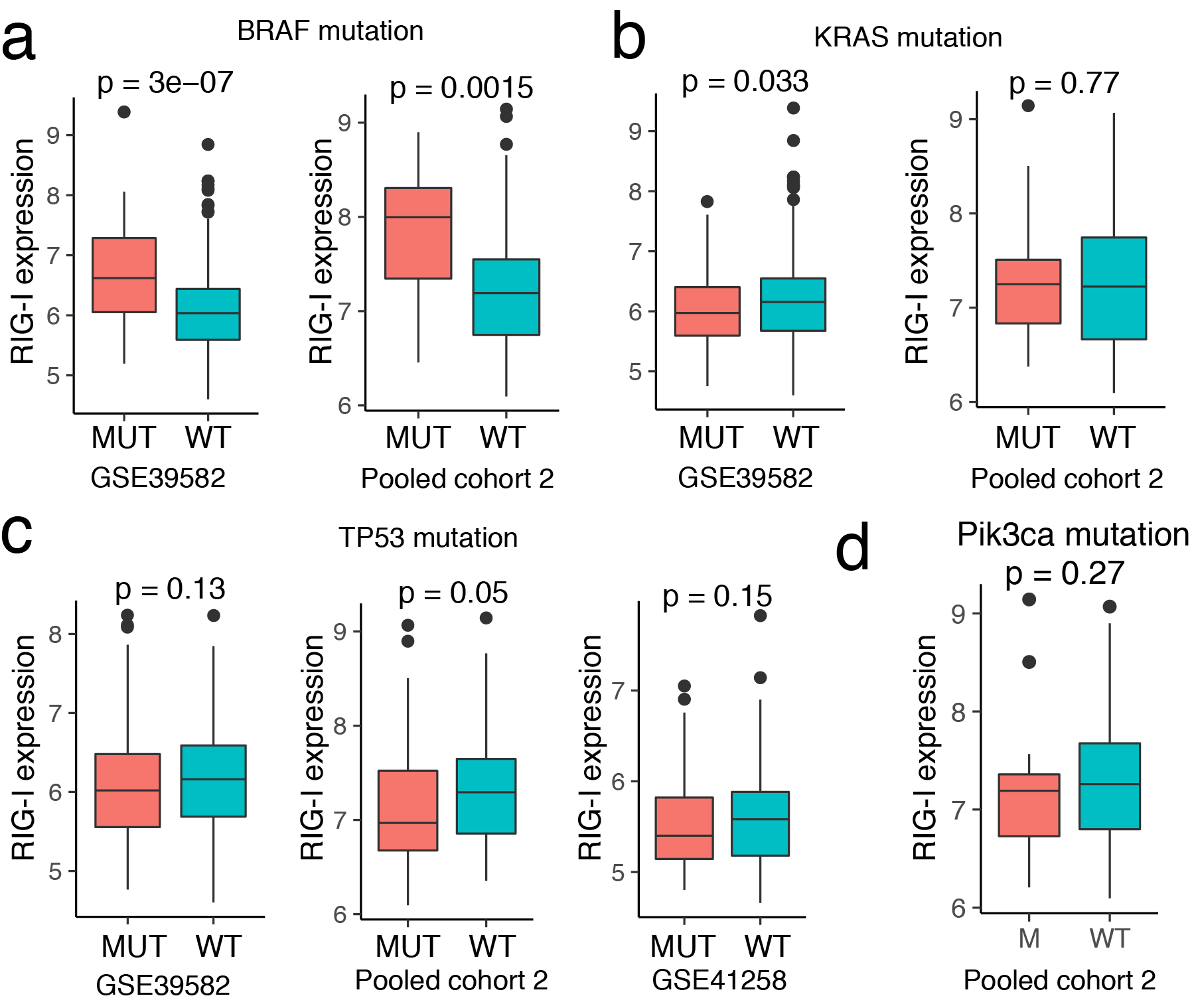
**

**Fig. S4:** Comparison of RIG-I expression between BRAF V600E, KRAS, TP53 and Pi3kca mutations in GSE39582, GSE41258 and Pooled cohort 2 cohorts.


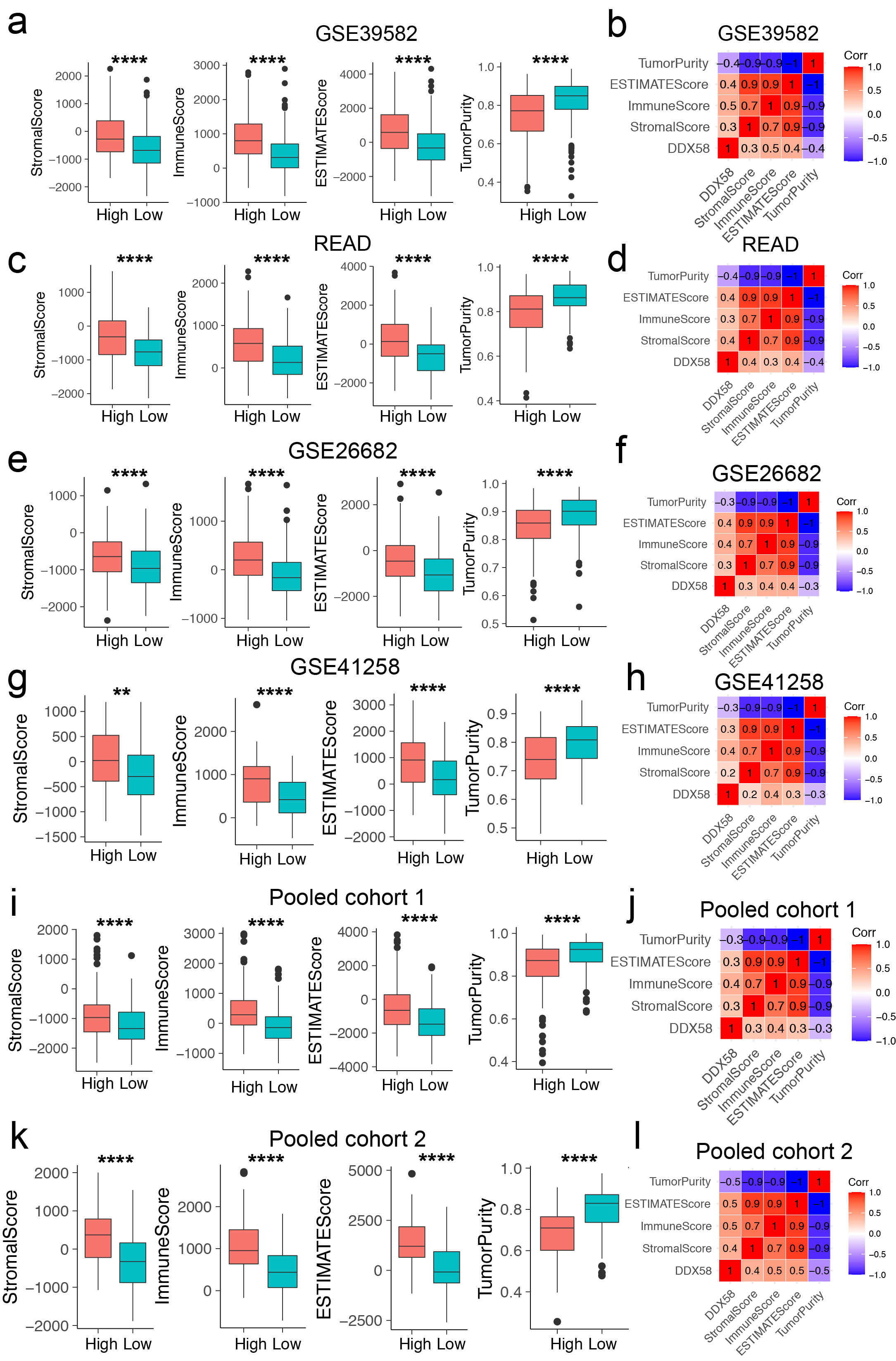


**Fig. S5:** The boxplot indicated the differences of immune score, stromal score, estimate score and tumor purity in six cohorts with high versus low RIG-I expression separated by median expression of RIG-I. The heatmap indicated the correlation between RIG-I expression and immune score, stromal score, estimate score and tumor purity in six cohorts with high versus low RIG-I expression separated by median expression of RIG-I.


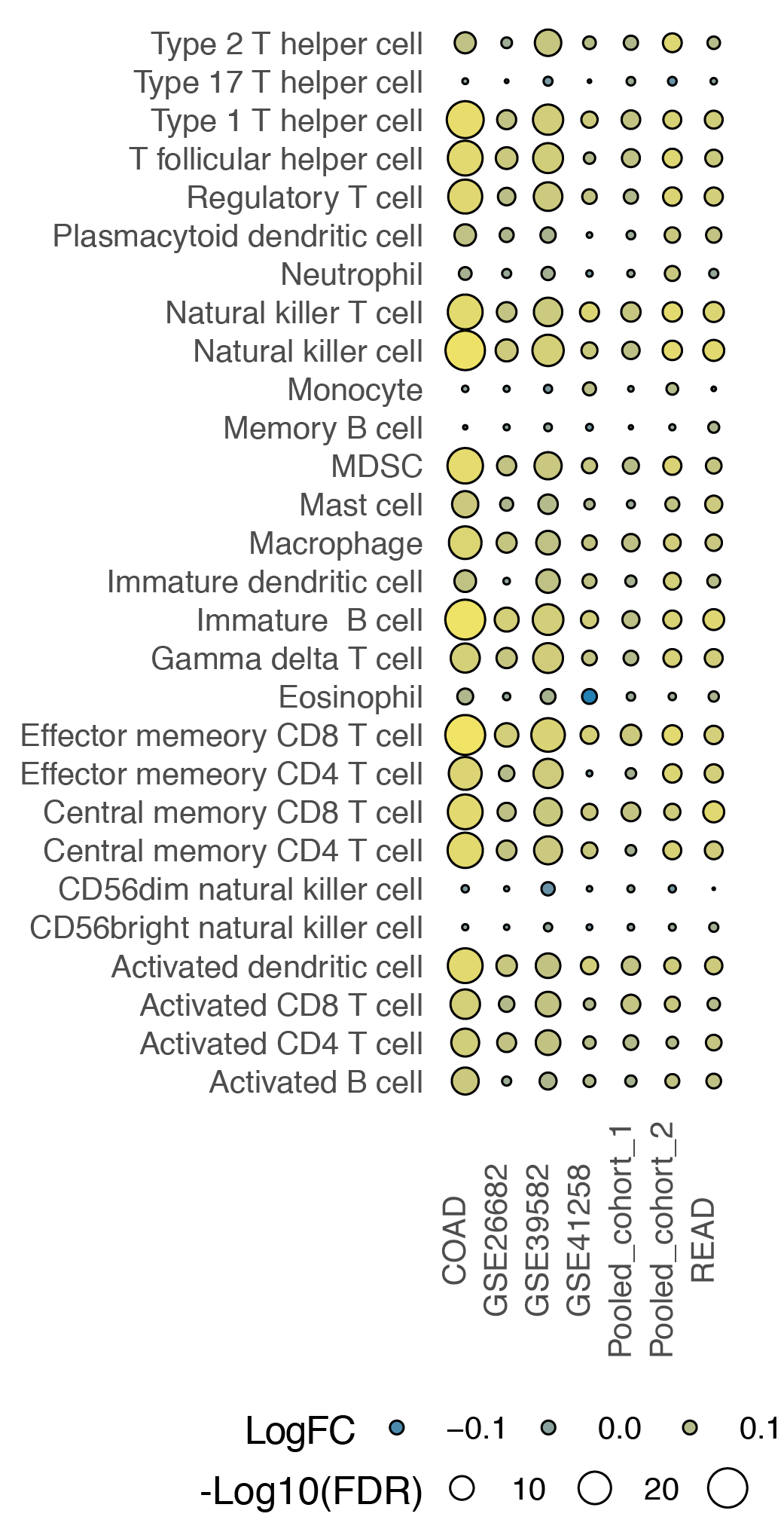


**Fig. S6:** Bubble plot representation showing the log2 fold change of immune cell infiltration in seven cohorts with high versus low RIG-I expression separated by median expression of RIG-I.

**
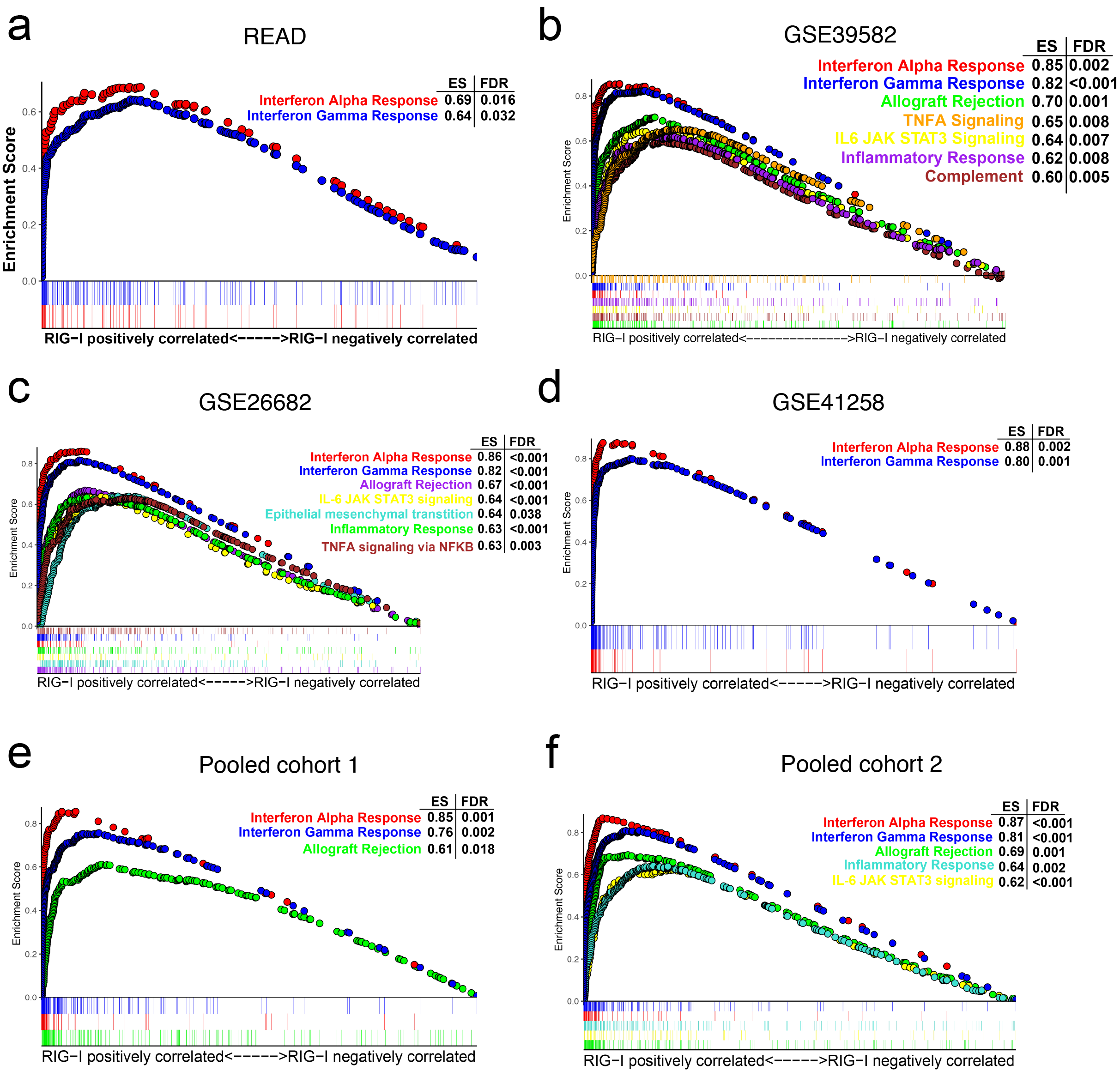
**

**Fig. S7:** Gene set enrichment analysis (GSEA) showed the significant functional gene sets enriched in Pooled cohort 1, Pooled cohort 2, READ, GSE39582, GSE26682, and GSE41258 cohorts with RIG-I highly expressed.


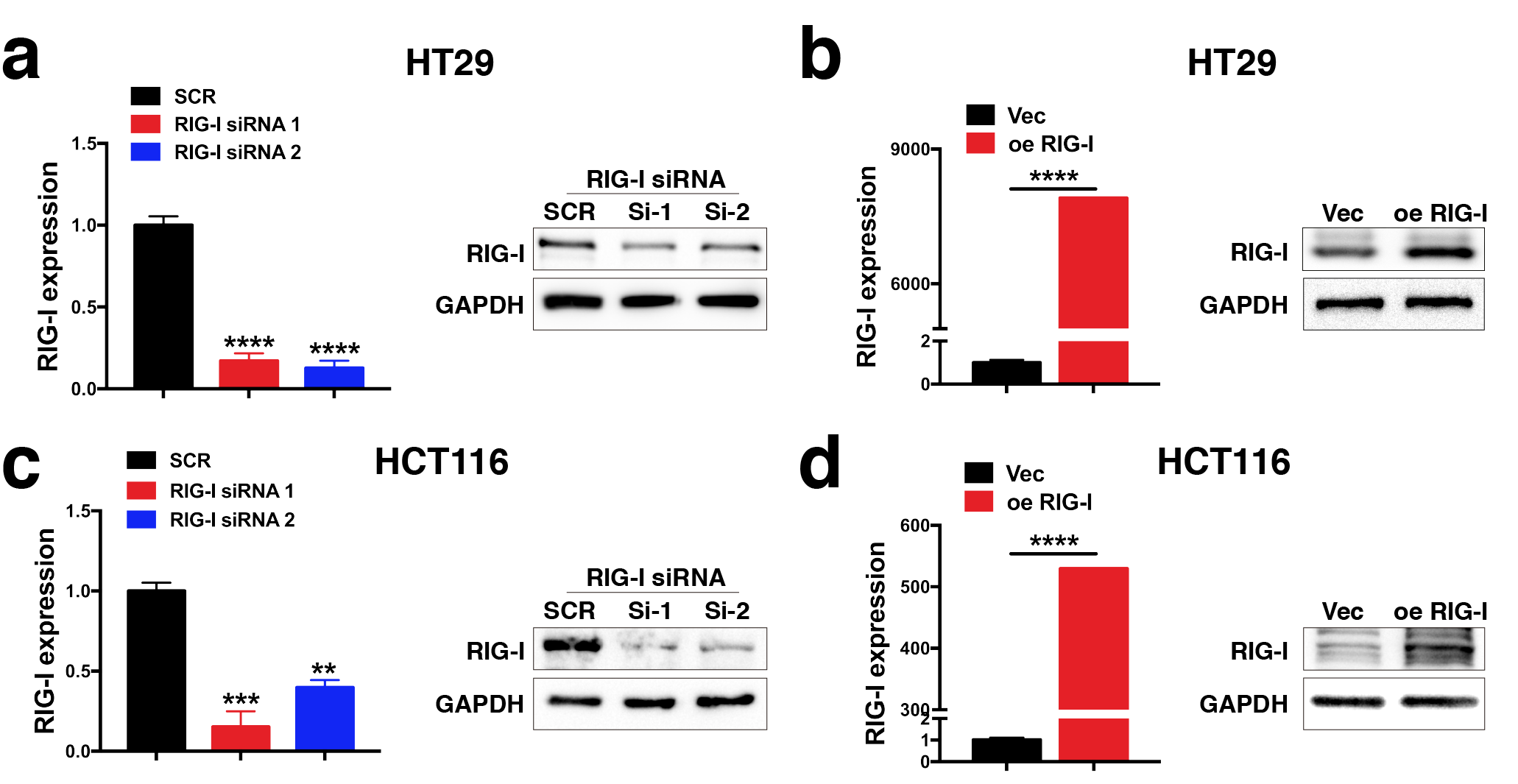


**Fig. S8: Transfection efficiency of RIG-I plasmid and siRNA in CRC.** Real‐time PCR (left) and western blotting (right) were used to analyze the mRNA and protein expression levels of RIG-I in HT29 cells **(a)** and HCT116 cells **(c)** transfected with SCR and siRNA (si-1, si-2). Real‐time PCR (left) and Western Blotting (right) were used to analyze the mRNA and protein expression levels of RIG-I in HT29 cells **(b)** and HCT116 cells **(d)** transfected with Vec and RIG-I plasmids (oe RIG-I). **P* < 0.05, ***P* < 0.01, ****P* < 0.001, *****P* < 0.0001 versus control group. SCR, scramble; Vec, vector.


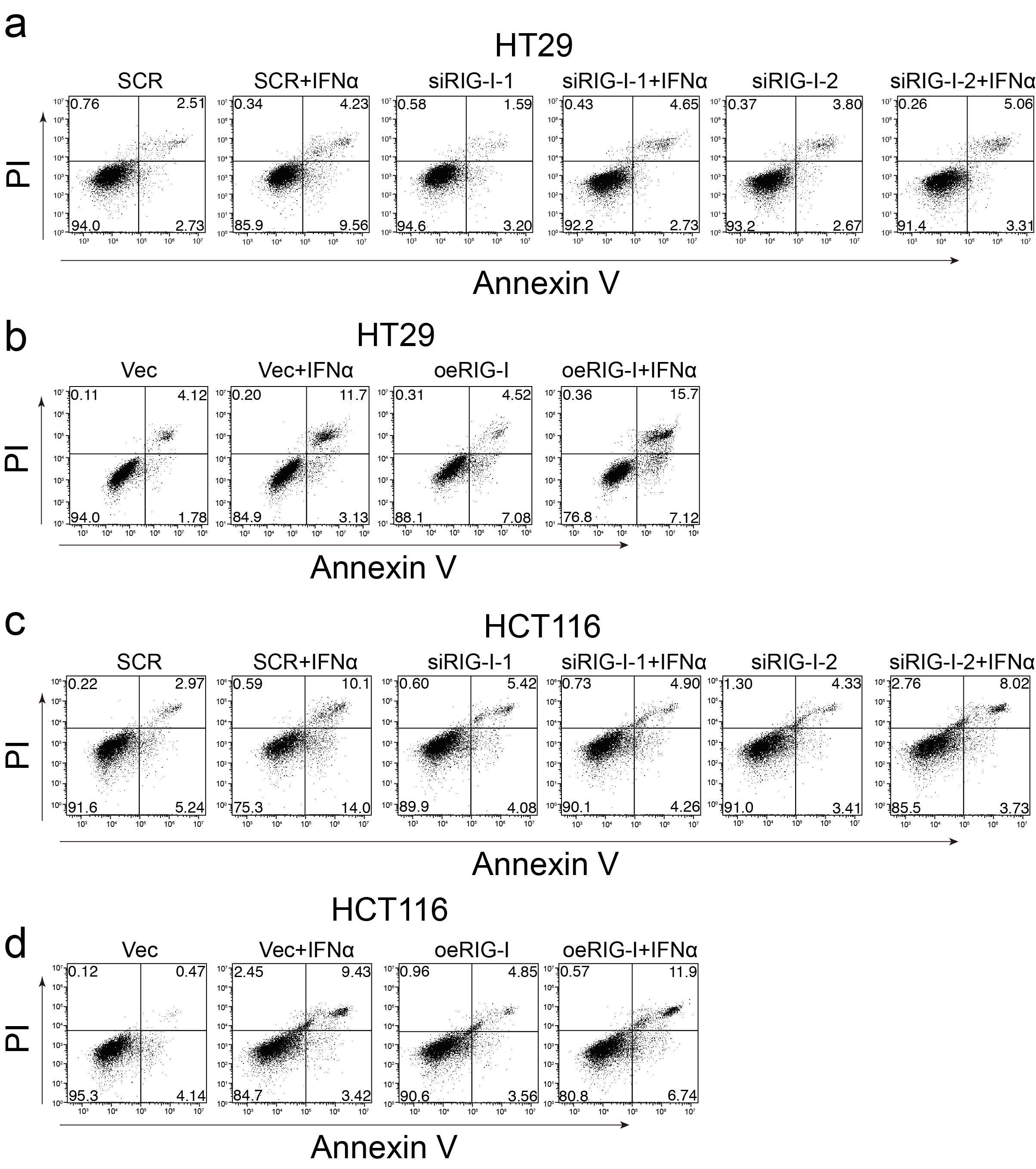


**Fig. S9:** HT29 and HCT116 cells were transfected with scramble and RIG-I small interfering RNA (siRNA) for 48 hours, and then treated with 2000 U/mL human recombinant IFN-α for 48 hours. Cells were isolated, stained with annexin V and PI and analyzed by flow cytometry. Quadrants from the lower left (counterclockwise) represent healthy, early apoptotic, late apoptotic, and necrotic cells, respectively. The only Annexin V-positive cells were regarded as apoptotic cells.

**
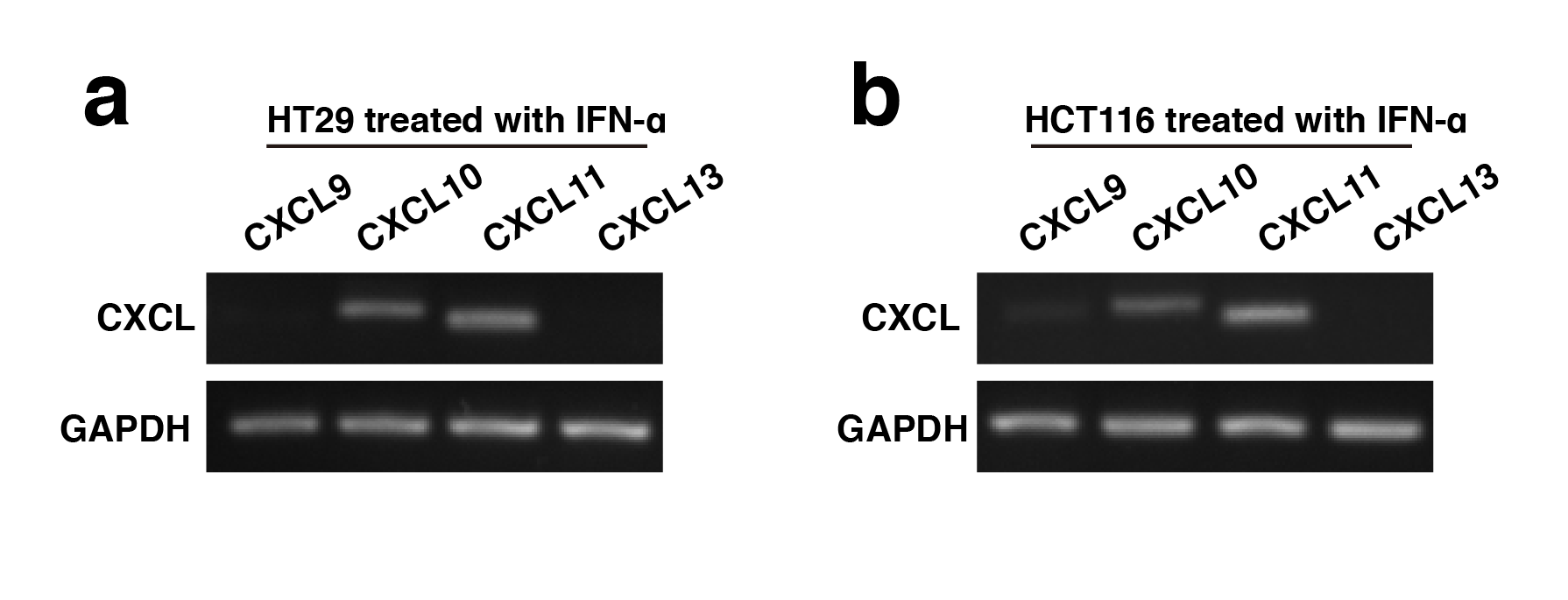
**

**Fig. S10:** The difference expression levels of CXCL9, 10, 11 and 13 under the interferon-α treatment in HT29 (a) and HCT116 cells (b) by PCR, respectively.


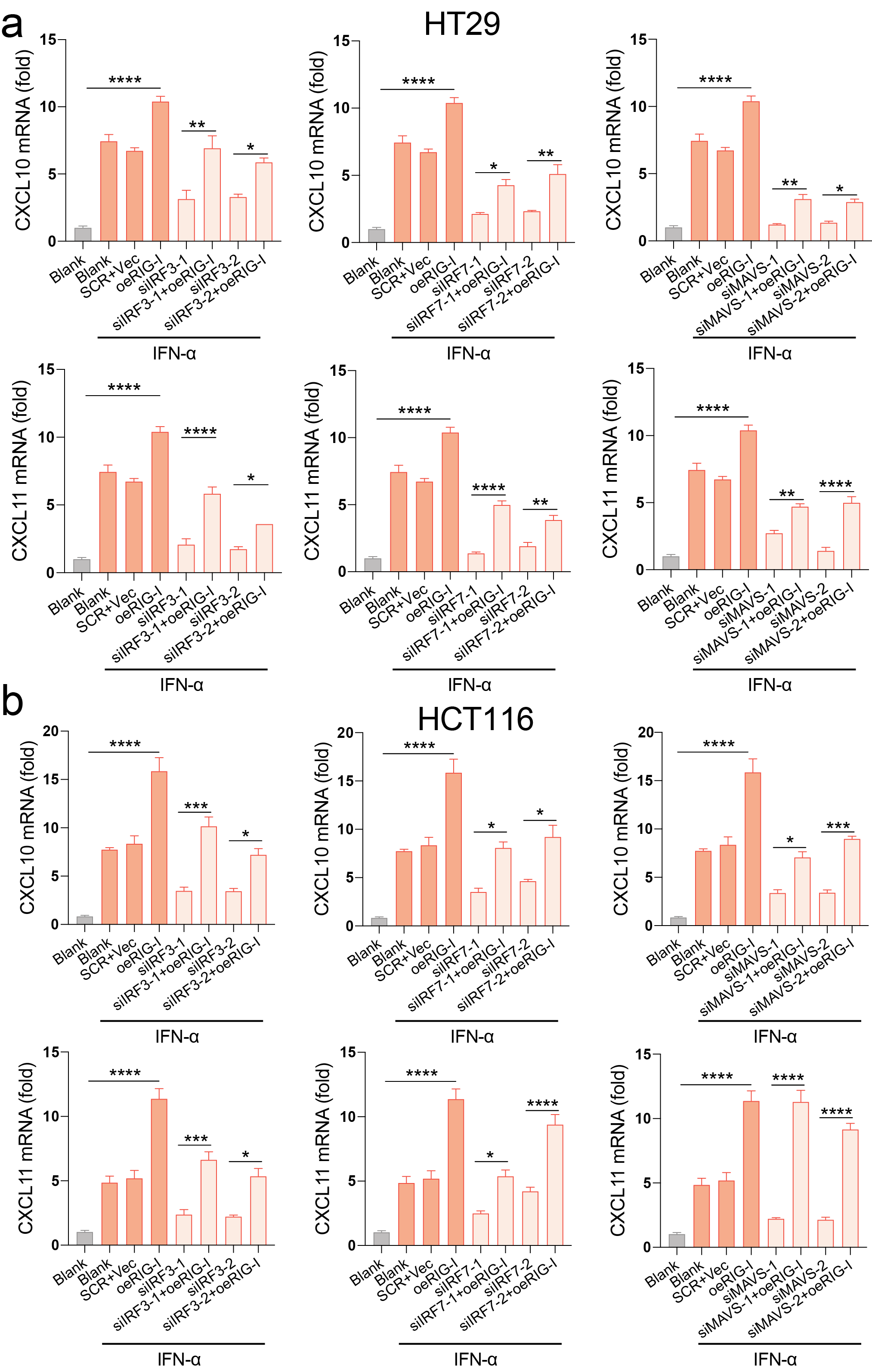


**Fig. S11:** HT29 (a) and HCT116 cells (b) were transfected with RIG-I overexpression plasmid, mitochondrial antiviral signaling protein (MAVS) siRNA, dependent interferon regulatory factor 3/7 (IRF3/7) siRNA for 48 hours, and then treated with 2000 U/mL human recombinant IFN-α for 48 hours. The CXCL10, CXCL11 expression were analyzed by RT-qPCR. **P* < 0.05, ***P* < 0.01, ****P* < 0.001, *****P* < 0.0001 versus control group.


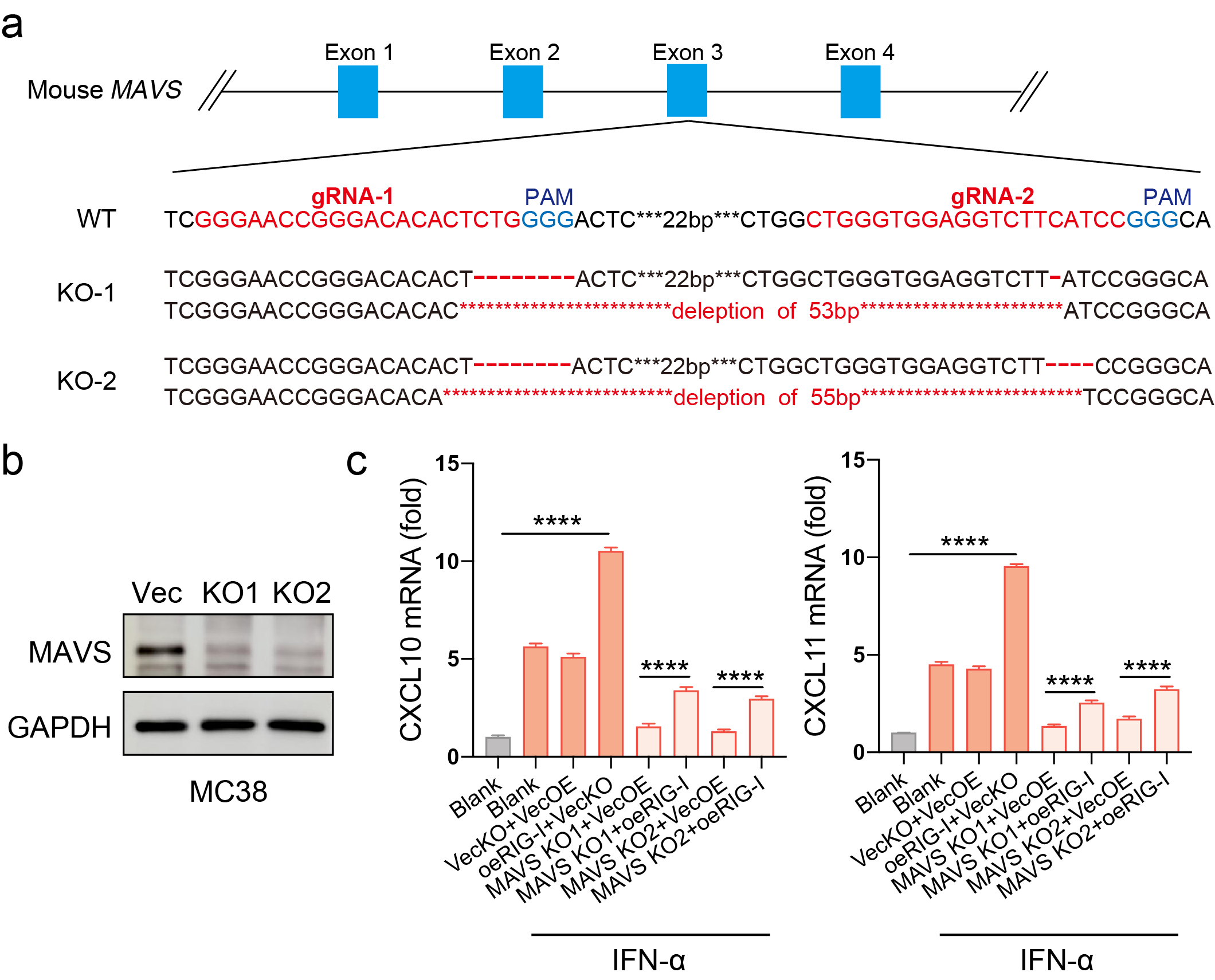


**Fig. S12: (a)** Top, a related portion of MAVS genomic structure. Bottom, sequences of the targeted region and the two knockout alleles (KO-1 and KO-2). Two guide RNAs (gRNAs) were used to achieve the targeting. **(b)** Transfection efficiency of two MAVS KO MC38 cells. **(c)** MAVS KO MC38 cells were transfected with RIG-I overexpression plasmid for 48 hours, and then treated with 2000 U/mL human recombinant IFN-α for 48 hours. The CXCL10, CXCL11 expression were analyzed by RT-qPCR. KO, knockout. *****P* < 0.0001 versus control group.

**
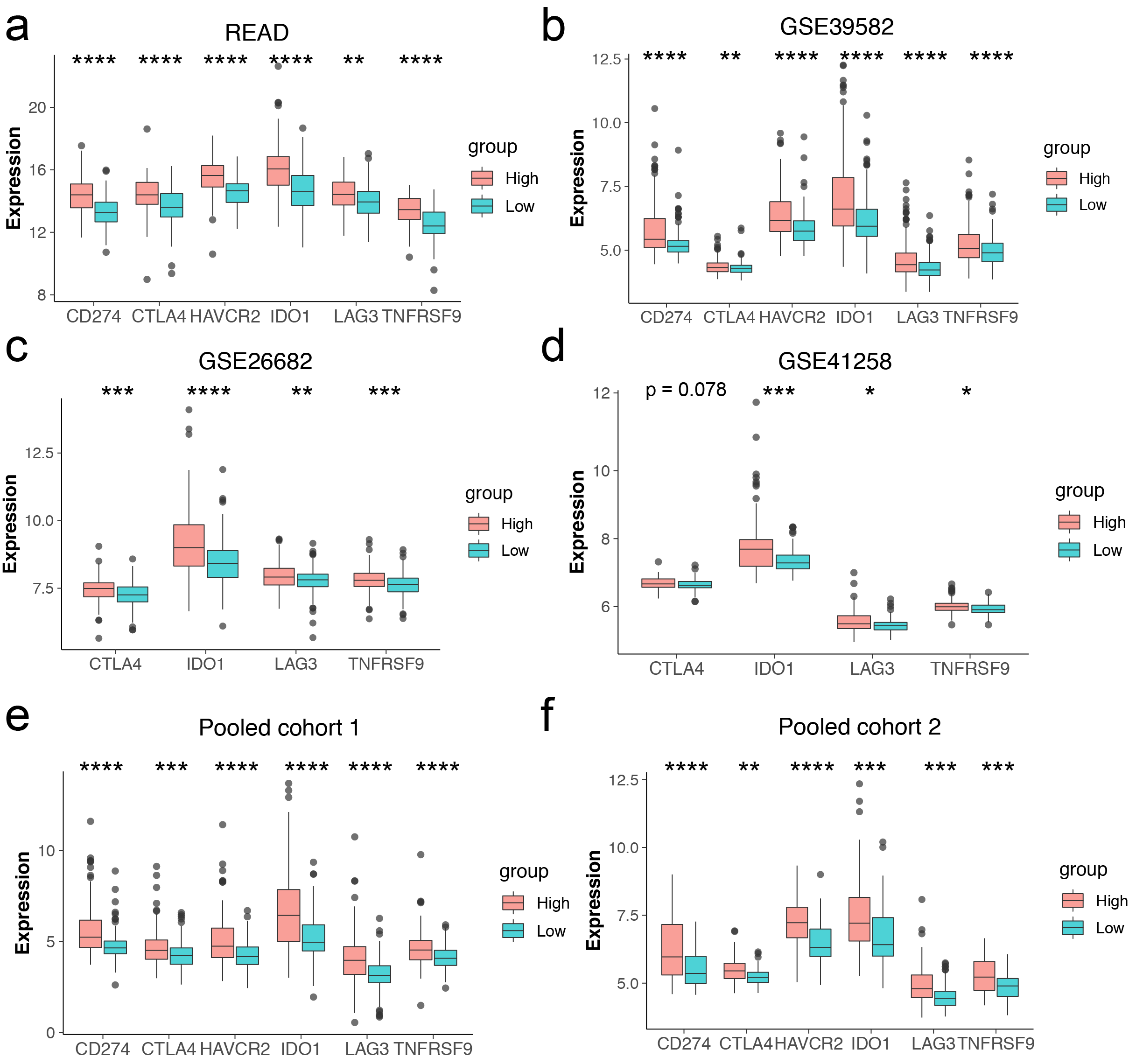
**

**Fig. S13:** The expression differences of immune-checkpoint genes, including LAG3, CD274, IDO1, HAVCR2, CTLA4 and TNFRSF9 in six cohorts with high versus low RIG-I expression separated by median expression of RIG-I.


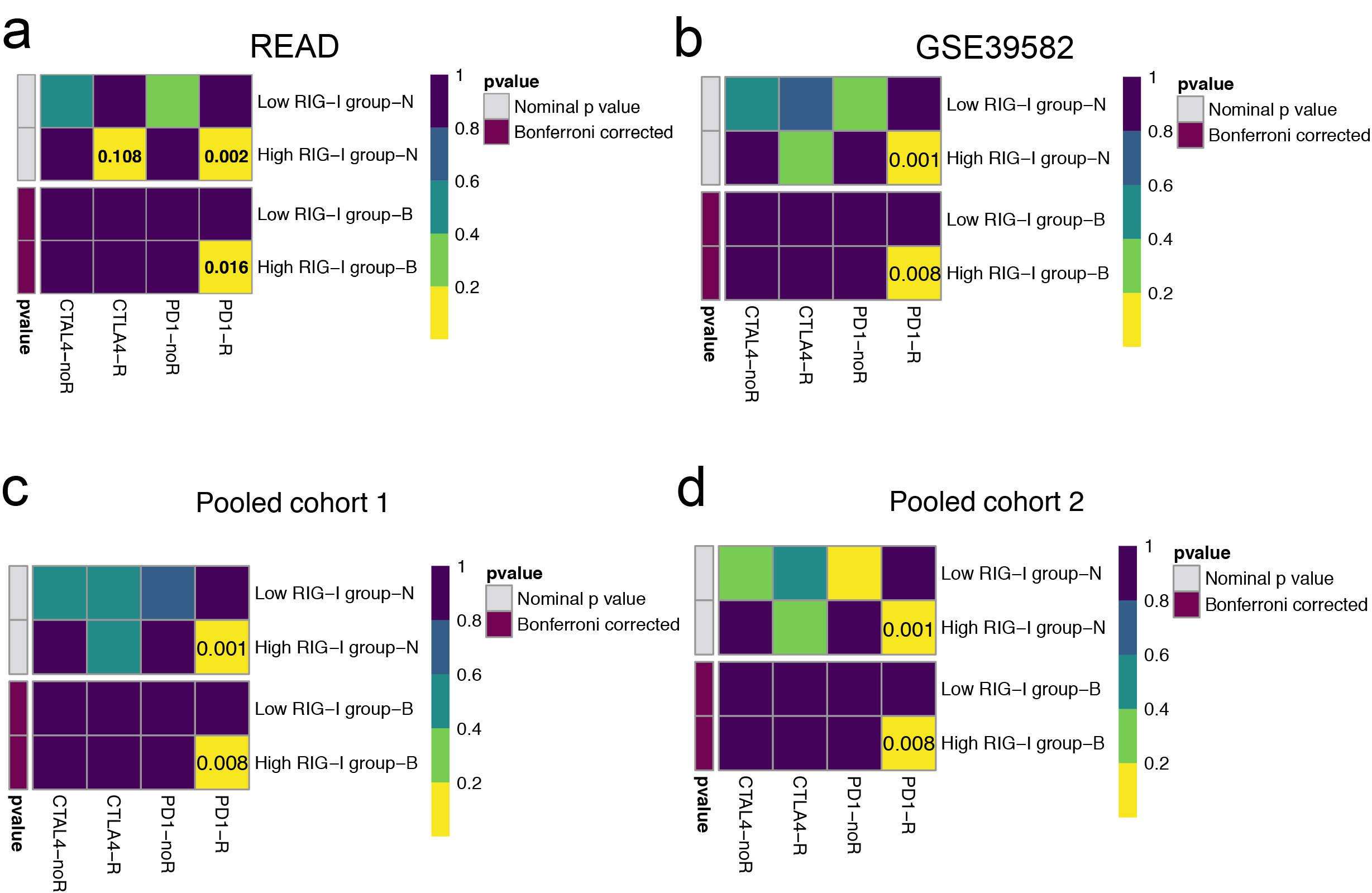


**Fig. S14:** High RIG-I expression may be more sensitive to the PD-1 inhibitor by SubMap analysis in Pooled cohort 1, Pooled cohort 2, READ and GSE39582 cohorts. **P* < 0.05, ***P* < 0.01, ****P* < 0.001, *****P* < 0.0001 versus control group.


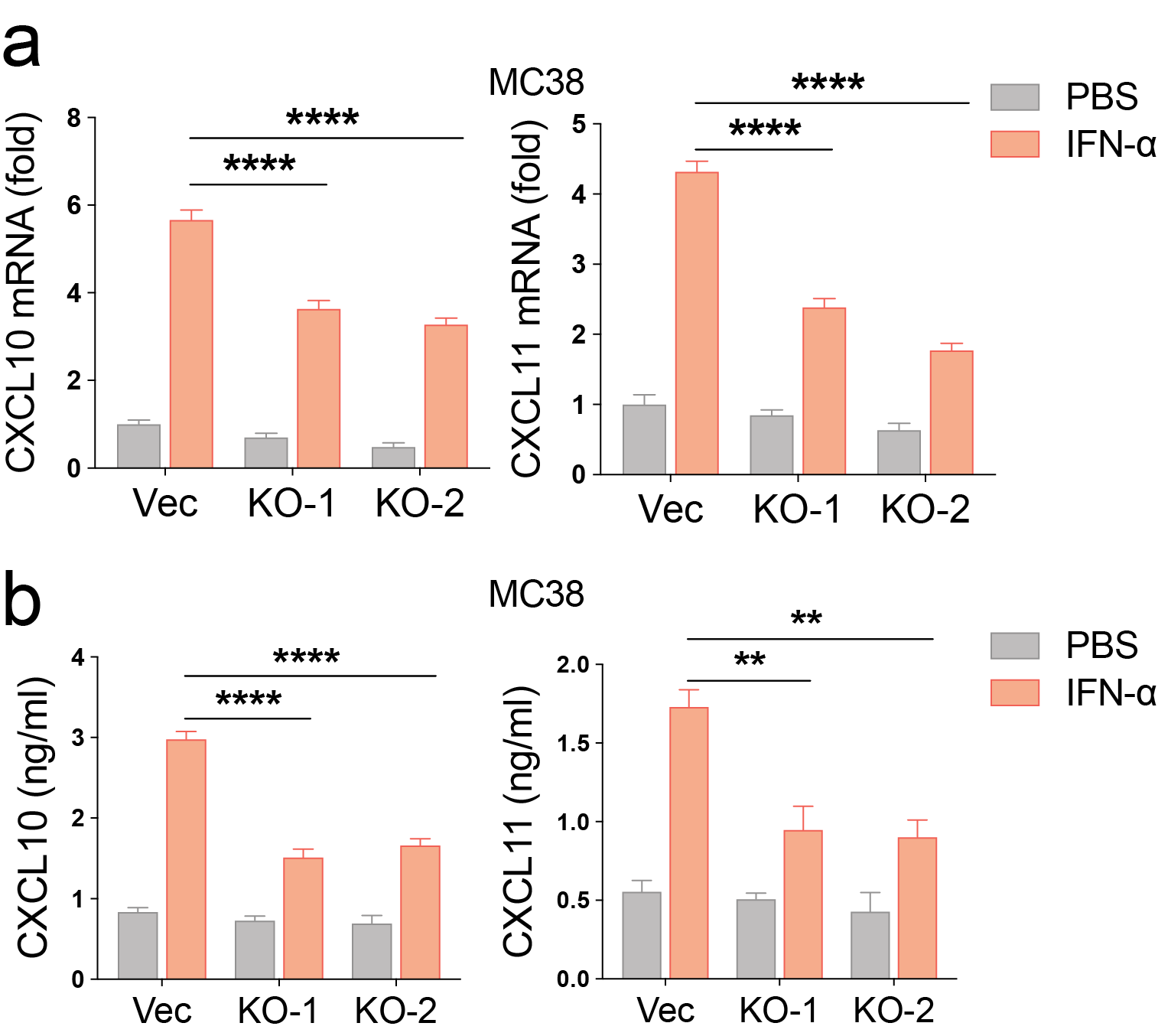


**Fig. S15: (a)** MC38 Vec and RIG-I knockout (KO) cells were treated with 200 ng/mL murine recombinant IFN-α for 48 hours. The CXCL10 and CXCL11 expression were analyzed by RT- qPCR. (**b**) Culture supernatants were collected after 48 hours treatment, and the protein levels of CXCL10 and CXCL11 were measured using ELISA. ***P* < 0.01, *****P* < 0.0001 versus control group.
