# Supplementary material for: Upregulation of RIG‐I is Critical for Responsiveness to IFN‐α Plus Anti‐PD‐1 in Colorectal Cancer
Source: Cancer Med. 2025 Mar 21;14(6):e70802. doi: 10.1002/cam4.70802 (PMC11926914; doi:10.1002/cam4.70802)
Supplement: Supplementary file 2 — Table S1. Clinical cohorts used in this study. [file CAM4-14-e70802-s007.docx]

| **Supplementary Table 1.**  **Clinical cohorts used in this study** | | |
| --- | --- | --- |
| **Cohort** | **Sample description** | **platform** |
| COAD | 455 CRC samples | Illumina HiSeq 2000 |
| READ | 167 CRC samples | Illumina HiSeq 2000 |
| GSE26682 | 331 CRC samples | GPL96 |
| GSE41258 | 166 CRC samples | GPL96 |
| GSE39582 | 566 CRC samples | GPL570 |
| Tissue microarray | 101 CRC samples | -- |
| Pooled cohort 1  (GSE4554+GSE13067+  GSE13294) | 313 CRC samples | GPL570 |
| Pooled cohort 2  (GSE39084+GSE35896) | 132 CRC samples | GPL570 |
