# Supplementary material for: Upregulation of RIG‐I is Critical for Responsiveness to IFN‐α Plus Anti‐PD‐1 in Colorectal Cancer
Source: Cancer Med. 2025 Mar 21;14(6):e70802. doi: 10.1002/cam4.70802 (PMC11926914; doi:10.1002/cam4.70802)
Supplement: Supplementary file 5 — Table S4. Sequences of primers for PCR. [file CAM4-14-e70802-s004.docx]

**Supplementary Table 4.** **Sequences of primers for PCR.**

| Gene |  | Real-time PCR |
| --- | --- | --- |
| Homo-GAPDH | 5’Primer | GTCTCCTCTGACTTCAACAGCG |
|  | 3’Primer | ACCACCCTGTTGCTGTAGCCAA |
| Homo-RIG-I | 5’Primer | CTGGACCCTACCTACATCCTG |
|  | 3’Primer | GGCATCCAAAAAGCCACGG |
| Homo-CXCL9 | 5’Primer | CTGTTCCTGCATCAGCACCAAC |
|  | 3’Primer | TGAACTCCATTCTTCAGTGTAGCA |
| Homo-CXCL10 | 5’Primer | GGTGAGAAGAGATGTCTGAATCC |
|  | 3’Primer | GTCCATCCTTGGAAGCACTGCA |
| Homo-CXCL11 | 5’Primer | AAGGACAACGATGCCTAAATCCC |
|  | 3’Primer | CAGATGCCCTTTTCCAGGACTTC |
| Homo-CXCL13 | 5’Primer | TATCCCTAGACGCTTCATTGATCG |
|  | 3’Primer | CCATTCAGCTTGAGGGTCCACA |
| Mus-GAPDH | 5’Primer | CATCACTGCCACCCAGAAGACTG |
|  | 3’Primer | ATGCCAGTGAGCTTCCCGTTCAG |
| Mus-CXCL10 | 5’Primer | ATCATCCCTGCGAGCCTATCCT |
|  | 3’Primer | GACCTTTTTTGGCTAAACGCTTTC |
| Mus-CXCL11 | 5’Primer | CCGAGTAACGGCTGCGACAAAG |
|  | 3’Primer | CCTGCATTATGAGGCGAGCTTG |
