# Supplementary material for: Upregulation of RIG‐I is Critical for Responsiveness to IFN‐α Plus Anti‐PD‐1 in Colorectal Cancer
Source: Cancer Med. 2025 Mar 21;14(6):e70802. doi: 10.1002/cam4.70802 (PMC11926914; doi:10.1002/cam4.70802)
Supplement: Supplementary file 6 — Table S5. Sequences of primers for CRISPR. [file CAM4-14-e70802-s006.docx]

**Supplementary Table 5.** **Sequences of primers for CRISPR.**

| Primers | | Sequences |
| --- | --- | --- |
| sgGFP | 5’→3’ | AGTTTTTACAAGGTCCAATC |
| RIG-I-mouse-sg1 | 5’→3’ | CCCCAGTTGGGGACCACTCACCA |
| RIG-I-mouse-sg2 | 5’→3’ | CCCAGTTGGGGACCACTCACCAT |
| RIG-I-mouse-sg3 | 5’→3’ | CCAGTTGGGGACCACTCACCATC |
