# Supplementary material for: Upregulation of RIG‐I is Critical for Responsiveness to IFN‐α Plus Anti‐PD‐1 in Colorectal Cancer
Source: Cancer Med. 2025 Mar 21;14(6):e70802. doi: 10.1002/cam4.70802 (PMC11926914; doi:10.1002/cam4.70802)
Supplement: Supplementary file 7 — Data S1. [file CAM4-14-e70802-s002.docx]

**2. METHODS AND MATERIALS**

**2.6. Immune-related bioinformatic analysis**

All CRC patients were divided into high and low expression groups based on the median of RIG-I expression. We first calculated the fraction of stromal score, immune score, estimate score and tumor purity in each group using the “estimate” R package [1]. Then, the immune infiltration landscape of CRC was constructed by calculating the infiltrating levels of the 28 immune cells in each CRC sample via a single-sample gene-set enrichment analysis (ssGSEA) algorithm (Table S3) [2, 3]. The “limma” R package was used to calculate the difference between the infiltrating cells in the tumors separated by median RIG-I expression (high versus low) [4]. The top 30 frequently mutated genes in different groups were analyzed using the “maftools” R packages [5].

SubMap analysis on the GenePattern website (https://cloud.genepattern.org) was used to indirectly predict the immunotherapy efficacy in patients in the high RIG-I expression group by measuring the similarity in gene expression profiles between our groups and melanoma patients treated with immunotherapies [6].

**2.7. Gene set enrichment analysis (GSEA)**

GSEA was used to enrich the significant gene sets between the high and low RIG-I expression groups according to the TCGA database. The H.all.v6.2 was downloaded from the Molecular Signatures Database and used as the reference set to represent well‐defined biological states or processes. A false discovery rate (FDR) of < 0.05 and |enrichment score (ES)| > 0.6 were used as the cutoff criteria.

**2.8. Patients, tissue microarray and immunohistochemistry**

Relevant clinical information, such as gender, age, TNM stage, and survival time, was also obtained (**Table S3**). The TM was assembled using a manual tissue puncher. A standard immunohistochemistry protocol was performed using an anti-RIG-I antibody (20566-1-AP; Proteintech). Pictures were acquired using an Aperio ImageScope software, and then, the histochemistry score was calculated using Halo3.0.311.314 (Indica labs, USA). The “survival” package was used to perform Kaplan-Meier survival analysis with a log-rank test. Additionally, This study was approved by the Ethics Committee of Zhongnan Hospital of Wuhan University (No. 2021032).

**2.9. Plasmids and siRNA transfection**

Human pcDNA3.1(+) and pcDNA3.1(+)-RIG-I plasmids were purchased from GenePharma (Shanghai). HT29 and HCT116 cells were transfected with pcDNA3.1(+)-RIG-I plasmids using Lipofectamine 2000 (Invitrogen, USA) to overexpress RIG-I. Vector pcDNA3.1(+) plasmid was used as a control.

RIG-I/MAVS/IRF3/IRF7 siRNAs were purchased from Ribobio (Guangzhou, China). HT29 and HCT116 cells were transfected with si-RIG-I-1 and si-RIG-I-2 using Lipofectamine 2000 (Invitrogen, USA) to knock down RIG-I expression. Scramble siRNA was used as a control. Sequences of siRNAs were as follows: si-RIG-I-1: TAGTAATGCTGGTGTAATT, si-RIG-I2: CCGGCACAGAAGTGTATAT; si-MAVS-1: CCATCCAAATTGCCCATCA, si-MAVS-2: CCATCCAAAGTGCCTACTA; si-IRF3-1: AGAGGCTCGTGATGGTCAA, si-IRF3-2: TCAGGGCCTTGGTAGAAAT; si-IRF7-1: CGAGCTGCACGTTCCTATA, si-IRF7-2: TCGAGTGCTTCCTTATGGA.

**2.10. Quantitative real-time PCR (qPCR) and semiquantitative PCR**

Total RNA was extracted from NCM460, HT29, HCT116 and SW620 cells using Trizol (Invitrogen, USA). One microgram of RNA was used to synthesize cDNA with a ReverTra Ace qPCR RT Kit (TOYOBO, Japan). Then, qRT-PCR was performed using SYBR-Green PCR Master Mix on a Roche LightCycler 96 PCR system. A semiquantitative PCR protocol was also used to determine the relative mRNA expression level (35 cycles). The human gene primers are shown in Table S4. Relative gene-expression quantification was quantified using the comparative CT method against GAPDH.

**2.11. Western blotting analysis and coimmunoprecipitation (CoIP)**

First, total protein fractions of CRC cells were obtained using NP40 lysis buffer (Beyotime, China). The BCA kits (Beyotime, China) were used to determine the concentration of the protein fractions. Then, 20 μg of protein from each group was run on an 8% SDS-PAGE gel and transferred to polyvinylidene fluoride (PVDF) membranes (Millipore, USA). The PVDF membranes were blocked with 5% fat-free milk and incubated overnight at 4 °C with the appropriate primary antibodies: RIG-I (381991, ZEN BIO, China), p-STAT1 (530453, ZEN BIO, China), STAT1 (381414, ZEN BIO, China), Caspase3 (#9662, CST, USA), Caspase7 (ab255818, Abcam, UK), SHP1 (201197, ZEN BIO, China), MAVS (#83000, CST, USA) and GAPDH (GB13002, Servicebio, China). Finally, enhanced chemiluminescence reagents (Thermo, USA) were applied to detect the bands after incubation with secondary antibody (GB23303, Servicebio, China). In addition, total protein fractions were collected for CoIP with anti-STAT1 antibody according to the manufacturer’s protocol for an IP/CoIP kit (Absin, China), and immunoblotting was used to analyze the protein interactions with the indicated antibodies. The quantification of STAT1 binding with RIG-I and SHP1 proteins under IFN-α treatment was compared using the ImageJ software.

**2.12. Cell apoptosis assay**

Cells in each group were double stained with annexin V and PI according to the manufacturer’s instructions (Bestbio, China). Then, the cells were analyzed via flow cytometry (BD FACSCanto II cell sorting system, BD Biosciences). Only Annexin V-positive cells were regarded as apoptotic cells.

**2.13. Isolation of tumor infiltrating lymphocytes (TILs) and flow cytometry**

Tumor samples were prepared from the tumors of MC38 cell-bearing mice via mechanical separation, and treated with collagenase P (2 mg/ml, Sigma) and DNase I (50 μg/ml, Sigma) for 10 min at 37 °C. To detect the frequency of CD3+ and CD8+ T cells, the cells were stained with anti-CD3 (Biolegend, USA), anti-CD45 (Biolegend, USA) and anti-CD8 (Biolegend, USA) antibodies with 1:100 dilution in PBS with 1% FBS. After incubation on ice for 30 min, flow cytometry data were obtained with a BD FACSCelesta cell analyzer (BD, USA) and analyzed using FlowJo software.

**2.14. Enzyme-linked immunosorbent assay (ELISA)**

The culture supernatants of CRC cells were used to quantify the protein levels of CXCL10 and CXCL11 using ELISA kits (eBiosence, CA, USA) according to the manufacturer’s instructions.

**2.15. Biolayer interferometry**

Recombinant human STAT1 (Abcam, ab82610) and human RIG-I (Abcam, ab132502) were purchased from Abcam. Protein interactions were measured and analyzed with an Octet Red instrument (Pall Corp.). Octet amine reactive second-generation (AR2G) biosensors were dipped into solutions containing STAT1 (1μg) and subsequently loaded with various RIG-I protein solutions. The protein association and disassociation processes were monitored and analyzed using Octet software and processed and graphed with GraphPad software.

References

(1) Yoshihara K, Shahmoradgoli M, Martínez E, et al. Inferring tumour purity and stromal and immune cell admixture from expression data. Nature communications. 2013;4:2612.

(2) Hänzelmann S, Castelo R, Guinney J. GSVA: gene set variation analysis for microarray and RNA-seq data. BMC bioinformatics. 2013;14:7.

(3) Charoentong P, Finotello F, Angelova M, et al. Pan-cancer Immunogenomic Analyses Reveal Genotype-Immunophenotype Relationships and Predictors of Response to Checkpoint Blockade. Cell reports. 2017;18:248-62.

(4) Ritchie ME, Phipson B, Wu D, et al. limma powers differential expression analyses for RNA-sequencing and microarray studies. Nucleic acids research. 2015;43:e47.

(5) Skidmore ZL, Wagner AH, Lesurf R, et al. GenVisR: Genomic Visualizations in R. Bioinformatics (Oxford, England). 2016;32:3012-4.

(6) Roh W, Chen PL, Reuben A, et al. Integrated molecular analysis of tumor biopsies on sequential CTLA-4 and PD-1 blockade reveals markers of response and resistance. Science translational medicine. 2017;9.
